# Microbial Communities of *Cladonia* Lichens and Their Biosynthetic Gene Clusters Potentially Encoding Natural Products

**DOI:** 10.3390/microorganisms9071347

**Published:** 2021-06-22

**Authors:** Tânia Keiko Shishido, Matti Wahlsten, Pia Laine, Jouko Rikkinen, Taina Lundell, Petri Auvinen

**Affiliations:** 1 Institute of Biotechnology, University of Helsinki, P.O. Box 56, 00014 Helsinki, Finland; pia.k.laine@helsinki.fi (P.L.); petri.auvinen@helsinki.fi (P.A.); 2 Department of Microbiology, Faculty of Agriculture and Forestry, University of Helsinki, P.O. Box 56, 00014 Helsinki, Finland; matti.wahlsten@helsinki.fi (M.W.); taina.lundell@helsinki.fi (T.L.); 3 Finnish Museum of Natural History, Botany Unit, University of Helsinki, P.O. Box 7, 00014 Helsinki, Finland; jouko.rikkinen@helsinki.fi; 4 Organismal and Evolutionary Biology Research Programme, Faculty of Biological and Environmental Sciences, University of Helsinki, P.O. Box 65, 00014 Helsinki, Finland

**Keywords:** nonribosomal peptides, polyketides, small molecules, shotgun metagenomics, *Cladonia*, lichen

## Abstract

Lichens have been widely used in traditional medicine, especially by indigenous communities worldwide. However, their slow growth and difficulties in the isolation of lichen symbionts and associated microbes have hindered the pharmaceutical utilisation of lichen-produced compounds. Advances in high-throughput sequencing techniques now permit detailed investigations of the complex microbial communities formed by fungi, green algae, cyanobacteria, and other bacteria within the lichen thalli. Here, we used amplicon sequencing, shotgun metagenomics, and *in silico* metabolomics together with compound extractions to study reindeer lichens collected from Southern Finland. Our aim was to evaluate the potential of *Cladonia* species as sources of novel natural products. We compared the predicted biosynthetic pathways of lichen compounds from isolated genome-sequenced lichen fungi and our environmental samples. Potential biosynthetic genes could then be further used to produce secondary metabolites in more tractable hosts. Furthermore, we detected multiple compounds by metabolite analyses, which revealed connections between the identified biosynthetic gene clusters and their products. Taken together, our results contribute to metagenomic data studies from complex lichen-symbiotic communities and provide valuable new information for use in further biochemical and pharmacological studies.

## 1. Introduction

Lichens are complex microbial assemblages recently redefined as ‘self-sustaining ecosystems’ [1], involving filamentous fungal, green algal or cyanobacterial (or both), and additional prokaryotic and yeast-like fungal partners living in symbiosis [2]. Lichens are common worldwide in almost all terrestrial environments and exist in diverse morphological forms [3,4]. Animals such as reindeer graze on lichens during the winter [5] and some lichens have even been used as emergency food by humans in the past [6]. Lichens or lichen extracts have also been used to treat skin, respiratory, digestive, and obstetric and gynecological disorders since the fifteenth and sixteenth centuries in Europe [7]. Natural products produced within lichen thalli are indeed known to include compounds with antifungal, antibacterial, anti-inflammatory, antioxidant, and antiproliferative activities [8,9,10,11].

Recently, interest in the pharmacological use of lichens has increased with the greater awareness of the environmental problems related to synthetic medicines and emerging antibiotic resistance. Many lichen-associated bacteria may serve a protective role for their hosts through the synthesis of secondary metabolites (SM) with antimicrobial properties [12]. One example of a newly found bacterial compound from lichens is uncialamycin, which was isolated from a lichen-associated *Streptomyces* strain and exhibits potent antimicrobial activities in the low nanomolar range [13]. Another example is nosperin, which is produced by a lichen-associated cyanobacterium that belongs to a family of compounds originally described from symbiotic bacteria in beetles [14].

Overall, a great majority of the over 1000 different natural products thus far reported from lichens are produced by filamentous fungi, which represent the predominant symbionts (mycobionts) of lichen thalli [15]. Phenolic compounds, dibenzofurans, terpenes, depsides, and depsones are the most abundant classes of SM compounds produced by these lichen-forming fungi [16,17]. Polyketide synthase (PKS) multimodular enzymes are involved in the generation of many previously described SM natural products from lichen mycobionts. For instance, the orcinol depsidone grayanic acid is biosynthesised via a PKS gene cluster found in the genome of *Cladonia grayi* [18]. The PKS biosynthetic gene clusters that produce the dibenzofuran usnic acid and 6-hydroxymellein are present in *Cladonia uncialis*, including additional genes for tailoring enzymes [19,20]. A similar PKS gene cluster (*Nppks7*) in *Nephromopsis pallescens* has also been annotated to be involved in the synthesis of usnic acid [21]. Recently, six producers of usnic acid (*Usnea florida*, *Evernia prunastri*, *Alectoria sarmentosa*, *Cladonia metacorallifera*, *Rhizoplaca melanophthalma*, and *Lobaria pulmonaria*) were found to have similar biosynthetic genes as *Cladonia uncialis*, indicating that the last common ancestor of all these species possessed the usnic acid gene cluster, which has been lost by some related lichen-forming fungi [22]. An in-depth analysis of the *Cladonia uncialis* genome revealed 48 biosynthetic gene clusters distributed in PKS, nonribosomal peptide synthetase (NRPS), hybrid PKS–NRPS, and terpene synthase complexes [23]. Despite having a very versatile biosynthetic potential for producing many SMs [23], the mycobiont of *Cladonia uncialis* used in the aforementioned study seems to generally only produce usnic acid [19].

Abiotic factors, including climate and humidity, and various biological elements may affect the compound composition of lichen samples [24]. Many lichens and isolated lichen symbionts grow very slowly both in the field and in the laboratory [10,15,25]. Very few genomes and metagenomes of lichen-associated organisms are presently available in public databases, which further hinders progress in assigning natural products to specific biosynthetic pathways [15]. Several close-to-complete genomes of lichen-forming fungi were obtained using a metagenome skimming approach, in which the environmental sample was sequenced using low-coverage shotgun sequencing followed by *in silico* extraction of the genomes from the assembled data [26,27]. With this method, in addition to saving time needed for laboratory culture and isolation of microbes, genomes of unculturable lichen-associated microorganisms can also be obtained for study. The initial comparison of 15 lichen mycobiont genomes revealed considerable potential for the production of SMs, with the fungal genomes containing 27 to 80 biosynthetic gene clusters [26]. Even different species within the class Lecanoromycetes (Ascomycota), among which the fungal genus *Cladonia* is included, carry vast potential for the biosynthesis of SMs [27,28]. The family Cladoniaceae consists of approximately 500 fungal species classified into 17 genera [29,30] and includes the well-known reindeer lichens, a phenotypic grouping of fruticose species of the genus *Cladonia* [29].

A recent study of the biosynthetic potential of novel bacteria from soil identified in genomes constructed using metagenomics and metatranscriptomics data highlights the use of environmental samples for drug discovery [31]. In this study, we used amplicon and shotgun metagenome sequencing approaches to investigate the biosynthetic potential of lichens for natural product synthesis. We analysed the metagenomes and predicted metabolomes from six *Cladonia* specimens representing five different species collected from Southern Finland. The microbial communities, potential natural product biosynthetic pathways, and selected natural products were characterised from each lichen ecosystem. Furthermore, the potential biosynthetic gene clusters present in the assembled lichen metagenomes were compared to published genomes of *Cladonia* species. The broad potential for new natural compounds was identified by detailed *in silico* analysis of the multiple biosynthetic gene clusters found in the metagenomes of *Cladonia* lichens.

## 2. Materials and Methods

### 2.1. Lichen Sampling and Environmental DNA Extraction

Six *Cladonia* lichen specimens were collected in Southern Finland near the Helsinki metropolitan area in autumn 2017 (L1–L4 from Luukki forest site, 60°19′ N 24°41′ E on 19 September 2017; and L34, L35 from Tapiola urban area, 60°10′ N 24°47′ E on 7 October 2017). The samples were kept in sterilised paper bags at 4 °C for 1 to 7 days. The specimens were cleaned in the laboratory by mechanical removal of particles and cut to remove soil and other particles. The samples were snap frozen with liquid nitrogen, homogenised using pestle and mortar, and kept at −80 °C until further analysis. The DNA extraction method was adapted from previously published protocols [32,33]. The homogenised frozen lichen material (0.4 g divided into two lysing matrix E tubes, MP Biomedicals, Solon, USA) was mixed with 10% CTAB extraction buffer [33] using a FastPrep-24^TM^ 5G Instrument (MP Biomedicals, Solon, USA) at 6.0 m/s for 30 s twice and with 5 min incubation on ice between runs. The samples were centrifuged at 16,000× *g* for 5 min at 4 °C and the aqueous layer was mixed with 0.5 µL of chloroform, vortexed, and centrifuged as described earlier. Nucleic acid precipitation was performed using PEG 6000/1.6 M NaCl solution [33] and purified with AllPrep DNA/RNA Mini kit (Qiagen, Hilden, Germany). The yield and quality of DNA extracted was measured using NanoDrop 1000 (Thermo Fisher Scientific, Waltham, USA) and 2100 Bioanalyzer (Agilent Technologies, Santa Clara, USA). As the DNA extracted from sample L4 contained impurities, it was further purified using DNeasy PowerClean Pro Cleanup kit (Qiagen, Hilden, Germany).

### 2.2. Microbial Community Analysis

The environmental DNA was amplified using specific primers to obtain internal transcribed spacer 2 (ITS2) region (primers ITS4 and gITS7) [34] and partial 16S rRNA encoding gene V3-V4 region (primers 341F and 785R) [35] following previously described conditions [36,37]. Sequences were obtained using a MiSeq (Illumina, San Diego, CA, USA). Adapters, low-quality bases (<30), and short sequences (<150) were removed using Cutadapt v.1.9.1 [38]. For ITS2 sequences, primer sequences were also removed in the previous step. The classification of operational taxonomic units (OTUs) was assigned using mothur v.1.42.3 [39], with Silva 132 as reference for 16S and UNITE for ITS2 sequences. The UNITE database was adapted to include green algae and yeast-selected sequences (List 1 in Supplementary Materials). The analysis followed mothur’s Standard Operating Procedure (SOP) for MiSeq [37,40] and consisted of the following for ITS2: to make contigs (make.contigs), to remove ambiguous sequences with a maximum length of 500 bp and homopolymer length of 50 bp to avoid sequencing artifact (screen.seqs (maxambig = 0, maxlength = 500, maxhomop = 50)), to obtain unique sequences (unique.seqs), to generate a table in which the names of the unique sequences are placed in rows and the names of the samples/groups are placed in columns (count.seqs), to find potentially chimeric sequences (chimera.uchime), to remove the chimeric sequences from the fasta file (remove.seqs), to remove singletons (split.abund), to classify the sequences using the modified library from UNITE, the k-Nearest Neighbor algorithm (knn), BLASTn for search and to identify the most similar sequence (classify.seqs (method = knn, search = blast, numwanted = 1)), to calculate uncorrected pairwise distances between sequences and to construct a column-formatted distance matrix with a cutoff value in which OTUs with distances larger than 0.5 will not be saved (pairwise.seqs (cutoff = 0.5)), to cluster the sequences (cluster), to obtain representative sequences using a label of 0.03 that corresponds to 97% sequence identity (make.shared (label 0.03)), to find the consensus taxonomy for each OTU sequence (classify.otu), to obtain the most common sequence from each OTU (get.oturep), and to obtain the sequences in fasta format (bin.seqs). The ITS2 sequences were also analysed using DADA2 v1.12 [41] following the guidelines from the program’s pipeline workflow to confirm the classification of the main fungal partner in the lichen. Eukaryotic OTU00001 and OTU00002 were differently classified in mothur and DADA2 analyses and a phylogenetic tree aided in classification of these OTUs. Representative sequences classified as *Cladonia arbuscula* (OTU00002) and *Cladonia rangiferina* (OTU00001) during mothur analysis were aligned using MAFFT v7 [42] and used to construct a maximum likelihood phylogenetic tree using IQ-TREE v1.6.12 [43,44] with ultrafast bootstrap (-bb 1000) [45]. The methods were calculated by ModelFinder [46] and consisted of K2P for *Cladonia arbuscula* (OTU00002) and K2P + G4 for *Cladonia rangiferina* (OTU00001) phylogenetic trees. The phylogenetic trees were annotated using iTOL v.5.771 [47] and Inkscape (http://www.inkscape.org, accessed on 18 February.2021). Mothur analysis for 16S rRNA gene sequences consisted of make.contigs, screen.seqs (maxambig = 0, maxlength = 462), unique.seqs, count.seqs, align.seqs, screen.seqs, filter.seqs, unique.seqs, pre.cluster, chimera.uchime, remove.seqs, split.abund, classify.seqs, remove.lineage (taxon = Chloroplast-Mitochondria-unknown-Archaea-Eukaryota), dist.seqs (cutoff = 0.40), cluster, make.shared (label 0.03), classify.otu, get.oturep, bin.seqs. The 16S rRNA sequences were aligned with the Silva reference (align.seqs) and cut to obtain the V3-V4 region (screen.seqs). Further de-noise was performed, allowing one difference for every 100 bp of sequence (pre.cluster). In addition, non-bacterial sequences were removed (remove.lineage), a distance matrix was generated, and distances larger than 0.40 were deleted (dist.seqs). Sequences present in low abundance (0.005%, counting for 50 sequences) were filtered, as suggested previously [48]. The figures were drawn using Rstudio v. 1.4.1106. Unclassified reads at family rank level for ITS2 and 16S amplicon sequences were further analysed for their taxonomic assignment and phylogenetic relationship. These analyses provided knowledge about the microorganism that could be further explored. All OTU sequences assigned as “unclassified” during mothur analysis and which had at least 50 sequences were used to construct the supplementary figures with the abundances of the sequences and their phylogenetic history. A maximum likelihood phylogenetic tree was obtained using the HKY85 substitution method tree implemented in the Phylogeny.fr platform [49]. The sequences obtained were submitted to the European Nucleotide Archive (accession number PRJEB34718).

### 2.3. Metagenome Sequencing, Assembly, and Analysis

The lichen DNA extractions were used to construct DNA libraries using Nextera XT kit (Illumina, San Diego, USA) following the manufacturer’s instructions and sequenced on NextSeq500 (Illumina, San Diego, CA, USA) platform in a paired-end manner (R1 = 170 bp, R2 = 140 bp). The reads were trimmed using Trimmomatic [50] with the following parameters: paired end, phred33, illuminaclip 2:30:10, leading 10, trailing 3, headcrop 15, sliding window 3:20, and minlen 36. The raw sequence data were analysed in the metagenomics analysis server MG-RAST v.4.0.3 [51] using RefSeq, Greengenes, RDP, SSU, and LSU databases for taxonomic assignment, and EGG NOG, COG, KEGG Orthologs, and SEED subsystems databases for functional assignments. The paired-end sequences were assembled using SPADES v.3.12.0 [52] for metagenomics assembly (metaspades.py). Trimmed reads were aligned with the assembled contigs using Bowtie 2 v.2.4.2 [53] to observe if most of the reads were used in the assembling. The completeness of the contigs was assessed using BUSCO v5.1.2 [54] against their ascomycota_odb10 (creation date: 10 September 2020, number of genomes: 365, number of BUSCOs: 1706). Further assessment of the quality of the assembly was verified using QUAST v5.0.2 [55] with the genome of *Cladonia rangiferina* ATCC 18275 (ASM614605v1) as a reference, and MetaQUAST [56] using a combined genome reference calculated by the software. Obtained contigs were also analysed in fungiSMASH (antiSMASH v.6.0.0 alpha1 but enabling beta) [57] for the presence of SM biosynthetic gene clusters. Five isolated *Cladonia* lichen-forming fungal species with genomes available in public databases (*Cladonia grayi* (Clagr3), *Cladonia macilenta* KoLRI003786 (GCA_000444155.1), *Cladonia metacorallifera* KoLRI002260 (GCA_000482085.2), *Cladonia rangiferina* ATCC 18275 (ASM614605v1), and *Cladonia uncialis* Normore8774 (GCA_002927785.1)) were also analysed using fungiSMASH to compare the potential biosynthetic gene clusters with the current studied metagenomic data. Assembled metagenome contigs were also analysed using the bacterial standalone version of antiSMASH (v.5.0), as it uses a more appropriate prodigal gene finding search for metagenomic data that uses pre-calculated training files for gene prediction. In addition, we also analysed the previously mentioned *Cladonia* genomes for comparison purposes. The detected biosynthetic gene complexes from fungiSMASH were clustered and their protein-level similarities were evaluated with BiG-SCAPE (Biosynthetic Gene Similarity Clustering and Prospecting Engine)/CORASON (Core Analysis of Syntenic Orthologs to prioritise Natural Product-Biosynthetic Gene Clusters) [58] (auto mode). Type I PKSs detected from the fungiSMASH analysis (129 proteins) were analysed using AAI-profiler [59] to observe the taxonomic identity of these sequences. These sequences were aligned with selected proteins using MAFFT v7, and Trimal v1.4.1 [60] (-automated1) was used to remove poorly aligned regions. A phylogenetic tree was constructed using IQ-TREE v1.6.12 using ModelFinder (LG + R7), tree reconstruction, and ultrafast bootstrap (1000 replicates) (-bb 1000 -alrt 1000 -nt AUTO). The proteins available in public databases were selected based on previously described lichen metabolites and 100% similarity predicted by fungiSMASH (Methylphloroacetophenone synthase (MPAS) A0A0R8YWJ7.2; 6-methylsalicylic acid synthase BAA20102.2; Orsellinic acid synthase J4UHQ6.1 and CBF73505.1; melanin BGC0001265; 1,3,6,8-tetrahydroxynaphthalene BGC0001257; naphthalene BGC0001906; alternariol BGC0000013; pyranonigrin E BGC0001124; 6-hydroxymellein BGC0001489; monascorubrin BGC0000099; grayanic acid BGC0001266; cichorine BGC0000037; naphthopyrone BGC0000107; 1-nonadecene BGC0001160; 1-heptadecene BGC0001164) retrieved from NCBI or MIBiG [61]. The sequences obtained were also submitted to the European Nucleotide Archive (accession number PRJEB34718).

### 2.4. Extraction and Chemical Analysis of Lichen Natural Product Compounds

Frozen lichen samples (0.5 g) were mixed with methanol (1 mL) and 300 mg of 0.5-mm glass beads (Scientific Industries, Bohemia, NY, USA). These samples were homogenised three times at 6.5 m/s for 20 s and once for 30 s (FastPrep-24^TM^ 5G Instrument, MP Biomedicals, Solon, OH, USA) with 5-min incubation on ice between runs. The samples were centrifuged at 10,000× *g* for 5 min at 4 °C. Methanol extracts were filtered using 0.2 µm pore size syringe filters (Phenomenex, Torrance, CA, USA) and a 1 mL syringe. Filtered extracts were kept in vials (Phenomenex, Torrance, CA, USA) and stored at −20 °C until mass spectrometry analysis. Lichen samples (7–16 mg of material) kept at room temperature were mixed with 1 mL of acetone and 300 mg of glass beads and homogenised three times at 6.0 m/s for 40 s (FastPrep-24^TM^ 5G Instrument, MP Biomedicals, Solon, OH, USA) with 5 min incubation on ice between the runs. Samples kept at room temperature (L1-0.007 g, L2-0.016 g, L3-0.013, L4-0.07, L34-0.008, L35-0.014) and frozen (L1-0.063 g, L2-0.161 g, L3-0.054, L4-0.048, L34-0.03, L35-0.03) were also extracted using acetone to compare with the methanol extraction. The extraction was performed as described above using methanol.

An Acquity Ultra Performance Liquid Chromatography (UPLC) system (Waters, Milford, MA, USA) coupled with a Kinetex^®^ 1.7 µm C8 100 Å, LC Column 50 × 2.1 mm (Phenomenex) was used to analyse the lichen extracts (0.5 μL). The run in the UPLC consisted of flow rate 0.3 mL min^−1^ at 40 °C in the following gradient mode: solvent A was 0.1% formic acid diluted in water, while solvent B was 0.1% formic acid in an even mixture of acetonitrile and isopropanol (1:1). The run started from a linear gradient of 95% solvent A and 5% of solvent B and in 5 min the gradient finished to 100% solvent B (0% solvent A) [62]. A UV–VIS diode-array detector identified the separation of the compounds and a Waters Synapt G2- Si mass spectrometer (Waters, Milford, MA, USA) recorded the mass spectra information. Positive and negative resolution modes in the electrospray ionisation (ESI) were used and precursor ions from *m*/*z* 100 to 2000 (scan time of 0.1 s) and product ions from *m*/*z* 50 to 2000 (scan time of 0.2 s) were recorded [62]. Other setup conditions consisted of capillary voltage of 1.5 kV in positive and 2.0 kV in negative ionisations; source temperature 120 °C while desolvation occurred at 600 °C; sampling cone 40.0; source offset 80.0; desolvation gas flow 1000 L h^−1^; and nebuliser gas pressure 6.5 bar [62]. Leucine–encephalin was used as an internal reference while sodium formate and Ultramark 1621 were used for calibration of the mass detection [62].

The analysis of the fungal compounds was performed using the UNIFI scientific information system (Waters, Milford, MA, USA). Positive and negative adducts (MH^+^, MNa^+^, and MH^−^) were compared against a library of 373 lichen-related compounds (List 2 in the Supplementary Materials) [63] and SciFinder chemistry database (https://www.cas.org/products/scifinder, accessed on 18 May 2020). The total ion chromatogram (TIC) and ultraviolet–visible light (210–800 nm) absorbance spectra chromatograms were also manually inspected to ensure that all major peaks were identified. Commercial references of usnic acid (Cayman Chemical Company, Ann Arbor, MI, USA) and atranorin (Carbosynth Ltd., Compton, United Kingdom) were dissolved in acetone to a final concentration of 0.1 mg mL^−1^. Atranorin was already fragmented in the ion source; therefore, fragment ions *m*/*z* 179.03389 (C_9_H_7_O_4_^+^) and *m*/*z* 163.04007 (C_9_H_7_O_3_^−^) were used for identification. Identification of perlatoric acid [64], rangiformic acid [65], and 4-O-methylolivetoric acid [66] were confirmed in reference to data presented in the cited publications.

## 3. Results

### 3.1. Biological Diversity within Cladonia Thalli

**Figure 1:**
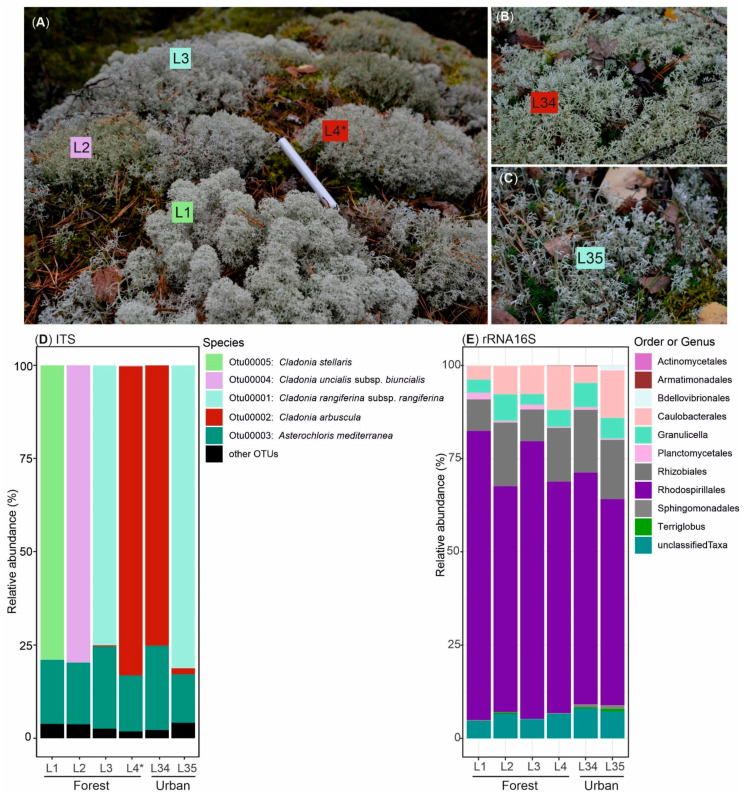
Lichens with the main fungal partner species belonging to the genus *Cladonia* included in this study. Photo of four *Cladonia* specimens collected in the boreal forest site (**A**) and two from the urban area (**B**,**C**). Communities of classified eukaryotes (**D**) and bacteria (**E**) obtained through analysis of amplicon DNA OTU sequences of the internal transcribed spacer 2 (ITS2) and 16S rRNA encoding gene V3-V4 (rRNA 16S) regions. * Sample L4 was later classified as *Cladonia mitis*.

*Cladonia* thalli were collected from boreal environments near Helsinki, Finland. Four different *Cladonia* species growing side-by-side on a rock outcrop at a boreal forest site (L1–L4, Figure 1A) and two additional specimens from a more urban area (L34 and L35, Figure 1B,C) were collected. The microbial communities living within the six specimens were studied based on targeted V3-V4 sequencing of the 16S rRNA encoding gene (for bacterial taxonomic OTUs) and the ribosomal ITS2 region (for fungal and green algae OTUs). The main aim when evaluating the microbial communities within the studied lichens was to observe which microorganisms could be present in these samples that would be further explored for their biotechnological potential. ITS2 data consisted of 1,324,861 raw reads. This was represented by 1,084,250 trimmed reads after removal of low-quality and short reads. These high-quality reads were assembled and assigned to 551 OTUs. However, to avoid overestimation of microbial diversity, sequences present in low abundance (<0.005% or 50 sequences) were filtered [48]. A total of 997,531 sequences distributed in 79 OTUs were further analysed, of which 81.7% were classified as fungi, and 18% were classified as plantae and 0.3% were classified as unknown sequences. From the fungal sequences, 98.6% were classified as Ascomycota (99.3% classified as Lecanoromycetes), Basidiomycota (0.6%), Chytridiomycota (0.1%), and unclassified (0.7%). Most of the fungal OTUs (814,774 sequences or 81.7%) corresponded to the main fungal partner of each lichen specimen, i.e., *Cladonia stellaris* L1, *Cladonia uncialis* L2, *Cladonia rangiferina* L3 and L35, and *Cladonia arbuscula* L4 and L34 (Figure 1D). Basidiomycetous yeasts previously detected in lichens [67] were not detected in abundance of OTUs above the filtered threshold. Other eukaryotic OTUs (Figure 1D) were less than 3% of the total reads and included the unclassified fungal OTU00006 (relative abundance of 0.4%) and the green algae *Trebouxia decolorans* (0.14%). Additionally, sequences from other organisms living in the surrounding environment were detected, such as the edible mushroom *Suillus variegatus* present in the forest samples (L1–L4) and the dung growing *Sphaerobolus stellatus* present in urban sample L35. Most of the green algae OTUs obtained from the thalli corresponded to *Asterochloris mediterranea*. In addition, ITS2 sequences corresponding to *Trebouxia decolorans/jamesii* were detected, but these were represented by less than 4% of the ITS2 OTUs in all samples (Supplementary Table S1).

The total 16S data of 1,297,148 raw reads were reduced by the quality process to 972,746 trimmed reads. These high-quality reads were analysed and further classified and divided into 158 OTUs. Most of the sequences (excluding 0.005% or 49 sequences possibly due to contamination) were assigned to Proteobacteria (88% or 95,171, in which 99.6% were classified as Alphaproteobacteria) and Acidobacteria (10% or 11,250). The bacterial community was similar in composition in all specimens and was mainly formed by the bacterial phyla *Proteobacteria* (orders *Rhodospirillales*, *Rhizobiales*, and *Caulobacterales*) and *Acidobacteria/Granulicella* (order *Acidobacteriales*). Other bacterial representatives formed a minority (Figure 1E).

In reference to the UNITE database, the ITS2 amplicon sequences of specimen L4 clustered with *Cladonia arbuscula*. However, the presence of rangiformic acid in this lichen contradicted with this species identification and instead pointed towards *Cladonia mitis*. Indeed, further analyses revealed that ITS2 OTU002 from this specimen had 98.44% sequence identity (100% coverage) with *Cladonia mitis* isolate O-L-184706 (accession number MK811894). This finding gained additional support from the maximum likelihood phylogenetic tree (Supplementary Figure S1A). Based on this evidence, we concluded that specimen L4 (OTU00002, Supplementary Table S2) belonged to *Cladonia mitis*.

The maximum likelihood tree showed that the ITS2 amplicon sequence that dominated in *Cladonia* samples L3 and L35 (OTU0001) belonged to *Cladonia rangiferina* or *Cladonia stygia* (Supplementary Figure S1B). While DADA2 grouped the amplicon sequence variants with *Cladonia stygia* (Supplementary Figure S2), the sequences were more similar to ITS2 of *Cladonia rangiferina* (Lendemer 46391, 99.61% identity; O-L195774, 98.83%; and Cl-200, 97.74%). Based on this evidence, we concluded that specimens L3 and L35 belonged to *Cladonia rangiferina*.

The diversity of taxonomically unclassified eukaryotic ITS2 (Supplementary Figures S3 and S4) and 16S rRNA (Supplementary Figures S5 and S6) V3-V4 region amplicon sequences was analysed based on their abundance in each lichen specimen and also considering phylogenetic relationships with previously sequenced lichen microbiota. Most of the unclassified ITS2 sequences, which corresponded to 1.35% of the analysed sequences, were distributed arbitrarily among the analysed lichen specimens. Nevertheless, OTU0006 was abundantly present only in *Cladonia stellaris* specimen L1 (Supplementary Figure S3). This fungal OTU belonged to the Leotiomycetes (Ascomycota), a diverse class known to also include lichen-parasitic fungi (Supplementary Figure S4). Most of the unclassified bacterial OTUs, which corresponded to 19% of the analysed sequences, belonged to Acidobacteria and Proteobacteria and were present in most lichen specimens studied (Supplementary Figures S5 and S6).

**Figure 2:**
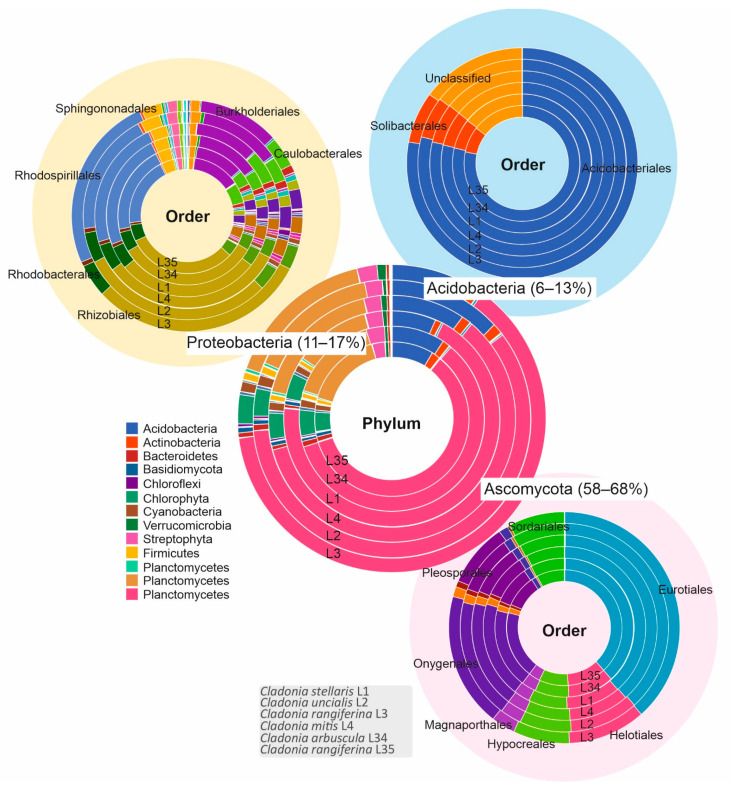
Taxonomic annotations of the metagenomics reads obtained using RefSeq best hit from the metagenomics analysis server (MG-RAST). The circle in the middle is larger and shows the most common phyla present in each sample (L1–L4, L34, L35) and their percentage range for three dominant phyla (Ascomycota, Proteobacteria, and Acidobacteria). Other phyla present in the samples are indicated in the colour legend beside the middle circle. The three smaller circles show the most common orders detected from Ascomycota (light pink), Proteobacteria (light yellow), and Acidobacteria (light blue).

In addition to ITS2 and 16S rRNA amplicon sequences for fungal and bacterial OTUs, we also obtained shotgun metagenome data from all six lichen specimens. BUSCO assessment showed that above 91% (L1-93.6%, L2-93.2%, L3-93.2%, L4-91.5%, L34-93.2%, and L35-93.4%) of the genes were complete when compared with the Ascomycota dataset (Supplementary Figure S7). Further assessment of the metagenome sequence quality can be observed using the genome of *Cladonia rangiferina* ATCC 18275 (ASM614605v1) as a reference (Supplementary Table S3) or multiple reference genomes (Supplementary Table S4). The total length of the metagenomes varied from 59 Mb (L4) to 94 Mb (L35) and the N50 varied from 2059 (L2) to 4902 (L4) (Supplementary Tables S3 and S4). While taxonomic assignments varied depending on the database used for classification by the metagenomics analysis server (MG-RAST [51]), most of the sequences were classified as Eukaryota (Supplementary Figure S8). The results based on searches against the NCBI RefSeq database indicated that most of the sequenced reads belonged to Eukaryota (65–76%), followed by Bacteria (22–34%), Archaea (0.1%), Viruses (0.01%), and other sequences (<0.003%) (Supplementary Figure S8). Most sequences belonged to the fungal phylum Ascomycota and to the bacterial phyla Proteobacteria and Acidobacteria (Figure 2). Interestingly, we did not observe a large number of sequences assigned to the Lecanorales order as expected, which could be due to limitations of MG-RAST in the analysis of eukaryotes. Among the annotated ribosomal RNA loci sequences of the order Eurotiales, most of the representatives belonged to the family Trichocomaceae (genera *Neosartorya*, *Aspergillus*, *Penicillium*, *Talaromyces*, and *Emericella*). 

Most of the bacteria-annotated sequences belonged to the orders already identified as OTUs. However, among the bacterial sequences identified in the phylum Proteobacteria, most were classified in the order Rhizobiales than in Rhodospirillales, which was contrary to the relative abundances of these bacterial orders in the 16S rRNA amplicon sequencing analysis.

Functional assignments were obtained for the metagenomic sequences and again there were differences depending on the reference database selected (Supplementary Figure S9). Although many functions remain to be determined, several protein-coding genes were assigned to participate in metabolism, information storage and processing, cellular processes, and signalling. Metagenomes from the six lichen specimens had similar protein functional assignments (Supplementary Figure S9).

### 3.2. Potential for Biosynthesis of Natural Products

The assembled contigs obtained from the six lichen metagenomes aligned with at least 85% of the trimmed reads (L1-89.63%, L2-86.46%, L3-88.87%, L4-85.06%, L34-89.13%, and L35-88.07%), which indicates that most of the reads were used to create the contigs. These contigs were compared with sequenced fungal genomes available in public databases (*Cladonia grayi* [68], *Cladonia macilenta* KoLRI003786 [69], *Cladonia metacorallifera* KoLRI002260 [70], *Cladonia rangiferina* ATCC 18275 (ASM614605v1), and *Cladonia uncialis* Normore8774 [19,20]) to identify genes putatively involved in the biosynthesis of SMs (Supplementary Tables S5 and S6, and Table 1). In total, 28 to 41 of such gene clusters were identified in each metagenome using fungiSMASH [58]; most represented type I PKS gene clusters (12–28, Table 1). *Cladonia* genomes contained 23 to 36 biosynthetic gene clusters, in which type I PKS corresponded to 12 to 19 (Table 1). The *Cladonia* metagenomes obtained in this study were also analysed using the standalone (antiSMASH_m) version of the search package (Supplementary Table S7).

A greater number of biosynthetic gene clusters were detected (in total 42 to 50) using the standalone version, which used the prodigal (prokaryotic gene recognition) method specific for metagenomic data (Supplementary Table S7). These detected genes could indicate bacterial biosynthetic pathways from the metagenomic data. However, *Cladonia* genomes were also analysed using the standalone version of antiSMASH and 3 to 15 more biosynthetic gene clusters were detected (Supplementary Table S7). These later samples represent genomic DNA sequenced from isolated fungi and this shows the differences in the results obtained depending on the web fungiSMASH (v6.0.0 alpha but enabling beta) and the standalone antiSMASH for metagenomic data (v5.0.0) versions.

The biosynthetic gene clusters detected by fungiSMASH were compared using BiG-SCAPE/CORASON [58]. Some of these clusters were found to be widely distributed among species of *Cladonia* (Supplementary Figures S10–S12). A hybrid cluster of NRPS and PKS biosynthetic genes (FAM00220, Supplementary Figure S10) showed high protein sequence similarity to PynA of the pyranonigrin E biosynthetic gene cluster (BGC0001124 in the MIBiG repository of known biosynthetic gene clusters [61]). However, the absence of a thioesterase domain in the lichen hybrid NRPS–PKS enzyme and differences in the surrounding enzyme encoding genes (PynBCDEFR are absent) of the putative pyranonigrin E biosynthetic gene cluster indicate that the compound produced by the enzyme complex may be different in the lichen thallus.

Enzymes involved in terpene biosynthesis (terpene synthases, TPSs) were present in many of our lichen metagenomes and in the sequenced *Cladonia* genomes and exhibited high protein sequence similarities with fungal farnesyl-diphosphate farnesyl transferase (FAM00157) and green algal phytoene synthases (FAM00311), which are involved in the synthesis of sterols and carotenoids, respectively (Supplementary Figure S11).

Type I PKS biosynthetic genes were widely detected in the searched fungal genomes and our lichen metagenomes, and a few of the genes were clustered (Supplementary Figure S12). A type I PKS (FAM00159) showed high protein-level similarity to a 1,3,6,8-tetrahydroxynaphthalene biosynthetic gene cluster (BGC0001257 MIBiG from *Nodulisporium* sp. ATCC74245) involved, for instance, in melanin biosynthesis. However, there were differences in the protein-coding domains, such as the absence of starter-unit acyltransferase (SAT) and second acyl carrier protein (ACP) domains among some of the biosynthetic genes in the *Cladonia* genomes and metagenomes (Supplementary Figure S12).

Non-reducing PKS biosynthetic gene clusters putatively involved in the synthesis of usnic acid were detected by antiSMASH (v5.0.0) for the lichen metagenomic data of *Cladonia arbuscula* (L34), *Cladonia mitis* (L4), *Cladonia rangiferina* (L3 and L35), *Cladonia stellaris* (L1), and *Cladonia uncialis* (L2) and in the sequenced genomes of *Cladonia grayi*, *Cladonia metacorallifera* KoLRI002260, *Cladonia rangiferina* ATCC 18275, and *Cladonia uncialis* Normore8774. However, fungiSMASH did not detect the usnic acid biosynthetic gene cluster, which is probably due to the fact that its sequence has been retrieved from the MIBiG database (February 2021).

**Figure 3:**
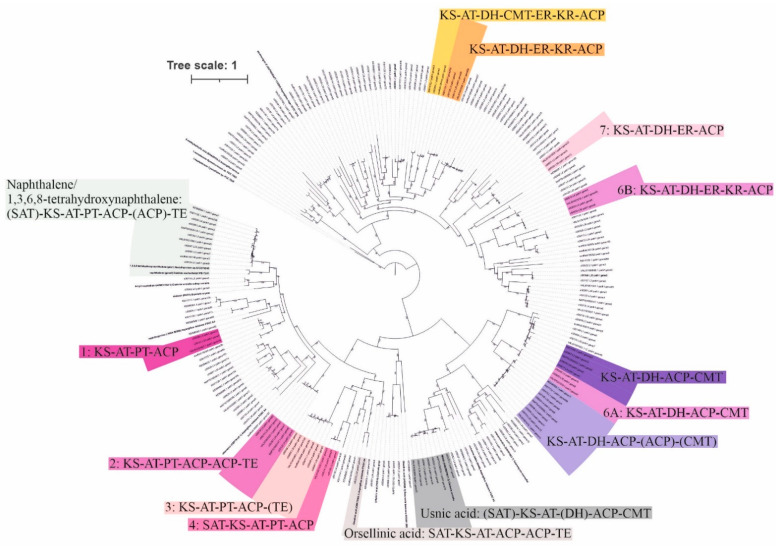
Phylogenetic history of type I PKS amino acid sequences present in *Cladonia* genomes and lichen metagenomes. Clades formed by PKS biosynthetic genes present in the three *Cladonia rangiferina* L3, L35, and ATCC 18275 are coloured in different shades of pink and indicated with numbers from 1 to 7. Previously described biosynthetic gene clusters are coloured in grey (usnic acid, orsellinic acid, and naphthalene/1,3,6,8-tetrahydroxynaphphalene). Four clades present in multiple samples are highlighted in yellow, orange, and light and dark purple. Parentheses indicate possible appearance of a domain. SAT: starter-unit acyltransferase; KS: ketosynthase; AT: acetyltransferase; PT: product template; ACP: acyl carrier protein; TE: thioesterase; CMT: C-methyltransferase; KR: ketoreductase; DH: dehydratase; ER: enoylreductase.

All type I PKS sequences (129) obtained from the fungiSMASH analysis of *Cladonia* genomes/metagenomes belonged to fungi, in which 32% were taxonomically assigned to *Cladonia* spp. (Supplementary Figure S13). A phylogenetic tree was constructed using these type I PKS sequences and others obtained from public databases (NCBI and MIBiG) (Figure 3). The detected putative usnic acid biosynthetic pathways were clustered, with the exception of *Cladonia uncialis* (L2), *Cladonia grayi*, and *Cladonia metacorallifera* KoLRI002260. A cluster previously observed in the BIGSCAPE/CORASON analysis (FAM00159) also formed a clade with 1,3,6,8-tetrahydroxynaphthalene and naphthalene biosynthetic gene clusters. *Cladonia rangiferina* L3 and L35 grouped with *Cladonia rangiferina* ATCC 18275 seven times (one of the pathways consisted of two minimally functional PKSs; number 6 in Figure 3). The domain composition of a selected type I PKS was based on the presence of a minimum domain composition for a functional enzyme, in which ketosynthase (KS), acetyltransferase (AT), and acyl carrier protein (ACP) were present [71]. Nevertheless, further analyses are necessary to demonstrate if the enzymes are actively involved in the synthesis of natural products.

### 3.3. Natural Products Detected in Lichen Thalli

**Figure 4:**
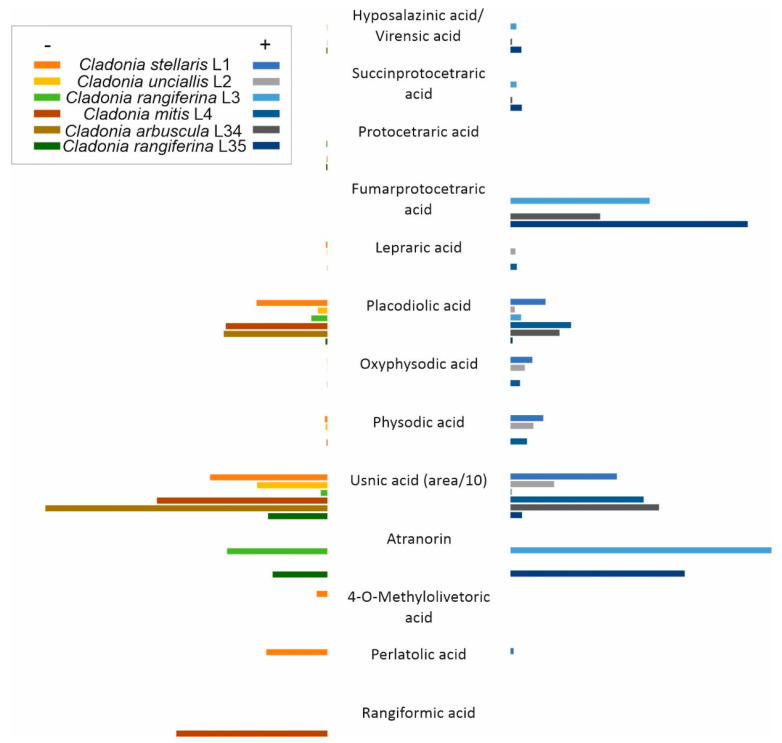
Relative amounts of lichen natural compounds fractionated by UPLC and identified by ESI-Q-TOF mass spectrometry. Compounds were detected using negative (left side of the figure, shown in yellow, orange, brown, and green bars) and/or positive (right side of the figure, shown in blue and gray bars) ionisation modes. Lichen extracts were obtained from specimens L1–L4, L34, and L35 kept at room temperature and extracted using acetone. Usnic acid was synthesised in higher amounts and the area value was divided by 10 to easily observe other compounds that were detected. Data visualised from Supplementary Table S8.

Extracts of the collected lichen specimens were fractionated by UPLC and analysed using an ESI-Q-TOF mass spectrometer to identify the SMs produced within the lichen thalli. The positive or negative modes of ionisation (or both) obtained by the mass spectrometer were used and a variety of lichen natural products were detected (Figure 4; Supplementary Figures S14 and S15, and Table S8). Compound prediction by the software UNIFI scientific information system (Waters, Milford, MA, USA) provided a rapid and assertive dereplication of known lichen compound molecular compositions, and the accuracy of prediction could be confirmed by analysing the commercially available references of atranorin and usnic acid. The lichen specimens contained aliphatic acids (rangiformic acid), chromones (lepraric acid), depsides (4-O-methylolivetoric acid, atranorin, and perlatolic acid), depsidones (furmarprotocetraric, protocetraric, succinprotocetraric, physodic, oxyphysodic, virensic, and hyposalazinic acids), and usnic and placodiolic acids (Figure 4; Supplementary Figures S14 and S15, and Table S8). We detected usnic acid in the negative and positive modes in all studied samples (Supplementary Figure S15). When investigating the ITS2 OTU amplicon sequencing data, we found that although the major fungal partner in the lichen specimens L3 and L35 was *Cladonia rangiferina* (Figure 1D), OTU sequences indicating other species (such as *Cladonia arbuscula*) were also present (Supplementary Table S2). Concurrently, we reanalysed the lichen specimens by using acetone solvent extraction prior to UPLC separation and MS analysis. In the acetone extracts of specimens L3 and L35, the predicted presence of the depside compound atranorin together with trace amounts of usnic acid was then detected (Figure 4).

## 4. Discussion

The lichenised fungal genus *Cladonia* has an almost cosmopolitan distribution and has been the target of many taxonomic and ecological studies over the years [29]. Recently, investigations on lichen microbial communities have expanded our knowledge of the diversity of eukaryotes and prokaryotes living in the lichen thalli [2,72,73,74,75,76,77,78,79,80,81,82,83,84,85,86,87]. Here, we used OTU amplicon sequencing of bacteria (16S rRNA V3-V4 gene region) and fungi/microalgae (ITS2 region) to observe the diversity of microbial communities present in the thalli of six *Cladonia* specimens collected from boreal forest and urban area environments in Southern Finland.

ITS2 OTU sequencing of the eukaryote community confirmed that the main mycobiont partner in all six specimens is represented by species of the lichenised fungal genus *Cladonia*, namely *Cladonia arbuscula*, *Cladonia mitis*, *Cladonia rangiferina* (two specimens), *Cladonia stellaris,* and *Cladonia uncialis*. Sample L4 was classified as *Cladonia arbuscula* by mothur analysis based on ITS2 amplicon sequencing. However, the phylogenetic analysis based on the representative sequence of ITS2 and the synthesis of rangiformic acid confirmed its assignment to *Cladonia mitis*. The genus *Cladonia* includes many species that are not easy to separate based on ITS region sequences [29,87], such as *Cladonia arbuscula* and *Cladonia mitis*. However, morphological and chemical analyses have been used to differentiate these two species [88].

The major photobiont/phycobiont partner in our lichen specimens formed a group with the green algae species *Asterochloris mediterranea*, which has been identified from thalli of other *Cladonia* species [79,89]. Most *Cladonia* species are known to be associated with the different species of *Asterochloris* or rarely with *Chlorella*, while species of *Trebouxia* are generally absent [29,89]. In our analysis, a small portion of the ITS2 sequences (4% or 6550 of the green algae sequences) grouped with *Trebouxia* spp. Even though a previous study correlated the presence of *Trebouxia* spp. in *Cladonia* with growth in a polluted site [90], we did not find significant differences in the low presence of *Trebouxia* in forest or urban *Cladonia* samples (Supplementary Table S1). This could be explained by ‘contaminant algae’ amplified from symbiont diaspores (soredia) of other lichen species invariably embedded into the felt-like outer surface of *Cladonia* thalli. This is despite the fact that multiple photobionts can sometimes be present within individual lichen thalli [49,91,92,93,94,95], and different algae have, for example, been found in basal (unclassified genera of Trebouxiophyceae and Ulvophyceae) or apical (*Asterochloris erici*) parts of *Cladonia squamosa* [83].

Most lichen-associated bacteria in the studied *Cladonia* specimens were Alphaproteobacteria (orders Rhodospirillales, Rhizobiales, and Caulobacterales) followed by Acidobacteria (order Acidobacteriales, genus *Granulicella*). The microbial community observed in the shotgun DNA sequencing metagenomes of the lichen specimens corresponded well with the amplicon analysis, in which most bacterial OTUs were represented by Proteobacteria and Acidobacteria. In previous studies, thalli of *Cladonia arbuscula* likewise were dominated by Alphaproteobacteria, as revealed by DNA staining and fluorescence in situ hybridisation (FISH) techniques [72,73,74]. A similar predominance of Proteobacteria followed by Acidobacteria and Actinobacteria was also reported for *Cladonia uncialis* and *Cladonia portentosa* [96]. Nevertheless, bacterial communities dominated by Acidobacteria (followed by Alphaproteobacteria, Gammaproteobacteria, Actinobacteria, Planctomycetes, and Verrucomicrobia) have been previously described for *Cladonia* thalli based on amplicon sequencing analysis [97]. Recent studies using high-throughput DNA sequencing of microbial communities have also confirmed the predominance of Alphaproteobacteria among the non-phototrophic bacteria in many other lichens [12,74,75,83,86,98,99,100].

While the microbial diversity of *Cladonia* lichens has been reported to depend on the mycobiont species [84], a lack of such specificity was found among *Cladonia mitis*, *Cladonia stellaris*, *Cladonia rangiferina*, and *Cladonia stygia* [86]. Furthermore, the latter study indicates the influence of geography in the diversity and abundance of bacteria living in reindeer lichens [86]. Taken together, our findings on the bacterial diversity within the six lichen specimens are consistent with these previous results and parallel DNA-sequencing-based community studies of *Cladonia* lichens. Our results indicate that the microbial community from samples collected in the boreal forest and urban area are similar, although we acknowledge the small amount of samples used in this study and that further analyses are necessary.

The bacterial community is thought to be involved in the supply of nutrients (mainly nitrogen, phosphorus, and sulphur), vitamins, and hormones for the main partners of the symbiosis, providing resistance to biotic and abiotic factors, and degradation of metabolites and senescent parts of the lichen thalli [98,101]. The most representative bacteria present in lichens include the order Rhizobiales (Alphaproteobacteria), which have the ability to perform nitrogen fixation and produce phytohormones and vitamins [102]. Other less representative bacteria, such as Rhodospirillales, were found in the lichen species *Lobaria pulmonaria* and proposed to be involved in potassium and nitrogen metabolism [12]. The study used a multi-omics-based approach, including metagenomics, metaproteomics, and metatranscriptomics data, to assign diverse functions for less representative lichen bacteria [12]. These divergent bacteria may aid in the metabolism of aromatic compounds, provide cofactors, vitamins, enzyme prosthetic groups and pigments, or mediate stress responses, virulence, disease, or defence (resistance to antibiotics and toxic compounds), among other functions [12].

Although most OTUs could be assigned to specific eukaryotic or prokaryotic taxa, a portion of our sequences remained unclassified at family rank level (1.35% for eukaryotic ITS2 and 19% for bacterial 16S rRNA V3-V4 gene region, respectively). The unclassified microorganisms should be further explored to elucidate their possible ecological functions in the lichen communities and their potential in the biosynthesis of natural products.

Many biosynthetic gene clusters putatively involved in the synthesis of SMs and natural products were detected in our lichen metagenomes (28 to 41 gene clusters) and in the published genomes of species of *Cladonia* (*Cladonia grayi* [68], *Cladonia macilenta* KoLRI003786 [69], *Cladonia metacorallifera* KoLRI002260 [70], *Cladonia rangiferina* ATCC 18275, and *Cladonia uncialis* Normore8774 [19,20]) (23 to 36 gene clusters). While over 1000 lichen metabolites were described, only three compounds were assigned to putative biosynthetic gene clusters [23]. Production of grayanic acid, a depsidone compound [18], usnic acid, a furandione compound [19], and 6-hydroxymellein, an isocoumarin compound [20] in *Cladonia* thalli was linked to specific PKSs. Nevertheless, further experiments are necessary to definitively link the products to their predicted biosynthetic pathways [23].

Comparative genomics based on protein homology searches of genome-sequenced fungi and assembled metagenomes of environmental samples have recently improved our knowledge of the biosynthesis of natural products, also in lichens [28,103]. Recently, the putative assignment of biosynthetic genes involved in the synthesis of grayanic acid, patulin, and betaenones A-C was performed for genome-sequenced *Cladonia uncialis* [103]. We detected similar biosynthetic gene clusters by using fungiSMASH and BiG-SCAPE/CORASON clustering analysis.

The phylogenetic history based on type I PKS sequences from *Cladonia* genomes/metagenomes and selected sequences available in the database shows a group formed with usnic acid producers, although the presence of SAT and DH domains could vary among the biosynthetic gene clusters. A previous study hypothesised the common ancestry of the usnic acid biosynthetic cluster genes, which has been lost from strains that can no longer produce this compound [22]. *Cladonia rangiferina* L3 and L35 produce atranorin, a β-orcinol derivative lichen compound. The biosynthesis of atranorin includes a PKS pathway to synthesise the orsellinic acid or methyl-3-orsellinate molecule [104,105]. An oxidase and a dehydrogenase may also be involved in the synthesis of the atranorin precursor haemmatomoyl aldehyde [105]. While the biosynthetic pathway of atranorin is still unknown, CrPKS16 may be involved in the biosynthesis of the atranorin precursors [106]. Three non-reducing PKS clades can be observed close to the orsellinic acid biosynthetic gene cluster from *Aspergillus nidulans* FGSCA4 and grayanic acid from *Cladonia grayi* (Figure 3). These three biosynthetic pathways include another highly reducing PKS gene, which is incomplete for clades number two and three. Furthermore, clades number two and three have other *Cladonia* genomes/metagenomes grouped together that do not produce atranorin (Figure 3). CrPKS16 (ALA65469.1) showed higher similarity only to one sequence of *Cladonia rangiferina* ATCC 18275 (VALI01000996.1) in a BLASTp search. Additional analyses are necessary to describe the atranorin biosynthetic gene cluster from *Cladonia rangiferina* L3 and L35.

Further analyses are necessary to confirm if the diversity of biosynthetic genes is due to their intrinsic differences, indicating that many compounds, if produced, may be specific for each fungal species or isolate. Moreover, expression of the predicted biosynthetic gene clusters in lichen fungi may be dependent on the microbiome composition together with abiotic factors and environmental stressors. In this study, we identified a remarkable diversity of biosynthetic gene clusters in the metagenomes of *Cladonia* thalli and detected aliphatic acids, chromones, depsides, depsidones, and usnic acid. Heterologous expression in tractable host organisms could improve our knowledge of the isolated and identified compounds produced by the *in silico*-predicted biosynthetic gene clusters. Recent development of heterologous expression platforms for fungal enzymes and enzyme complexes for biosynthesis of SMs [107] will likely facilitate the faster discovery of novel natural products from lichens and other symbiotic consortia of microorganisms.

## 5. Conclusions

The microbial communities living in six *Cladonia* lichen specimens were studied using 16S rRNA gene and ITS2 region amplicon sequencing for bacterial and fungal/microalgal partners, respectively, together with shotgun metagenomics. The bacterial community predominantly consisted of Alphaproteobacteria followed by Acidobacteria, while most of the eukaryotic sequences belonged to the main fungal partner (species *Cladonia arbuscula*, *Cladonia mitis*, *Cladonia rangiferina*, *Cladonia stellaris*, and *Cladonia uncialis*) and the green algal partner *Asterochloris mediterranea*. The metagenomics data revealed a large number of biosynthetic genes potentially involved in the synthesis of fungal secondary metabolite natural products (from 28 to 41 biosynthetic gene clusters in each lichen). In addition, our study also identified the compounds commonly produced by the studied lichens (such as usnic acid and atranorin) and the phylogenetic history of the polyketide synthases potentially involved in their synthesis. We combined high-throughput sequencing analyses to metabolomics in order to advance the research on natural products of lichens.

## Figures and Tables

**Table 1:** Potential biosynthetic gene clusters detected by fungiSMASH.

Sample	T1PKS	T3PKS	Terpene	NRPS	NRPS–T1PKS	T3PKS–T1PKS	Indole	Hybrid/T1PKS–Indole/Terpene	Siderophore	Other	Total
Metagenomes											
*Cladonia arbuscula* L34	20	2	3	7	3	0	2	1	0	0	38
*Cladonia mitis* L4	28	2	2	5	2	0	0	1	1	0	41
*Cladonia rangiferina* L3	19	1	4	6	3	1	0	0	0	0	34
*Cladonia rangiferina* L35	17	1	6	4	2	1	0	1	0	0	32
*Cladonia stellaris* L1	12	0	6	5	4	1	0	2	0	1	31
*Cladonia uncialis* L2	15	0	4	5	3	1	0	0	0	0	28
Genomes											
*Cladonia grayi*	12	0	2	9	1	0	0	0	0	1	25
*Cladonia macilenta* KoLRI003786	18	0	3	6	3	1	0	0	0	0	31
*Cladonia metacorallifera* KoLRI002260	19	0	5	8	1	1	0	1	0	1	36
*Cladonia rangiferina* ATCC 18275	18	0	2	6	2	1	0	1	0	0	30
*Cladonia uncialis* Normore8774	14	0	3	2	3	1	0	0	0	0	23

## Data Availability

The sequences obtained were submitted to the European Nucleotide Archive (accession number PRJEB34718).

## Supplementary Materials

The following are available online at https://www.mdpi.com/article/10.3390/microorganisms9071347/s1, Figure S1: Maximum likelihood phylogenetic tree constructed using representative ITS2 amplicon sequences of two operational taxonomic units OTU 00002 (A) and OTU00001 (B) obtained from mothur analysis, Figure S2: Microbial community at species taxonomic level inferred by DADA2 based on amplicon sequence variants (ASVs) from samples collected from a boreal forest (Luukki) and urban area (Tapiola) sites, Figure S3: Number of unclassified ITS2 sequences at family taxonomic level (*n* > 50) per sample, Figure S4: Evolutionary history based on ITS2 unclassified operational taxonomic units (OTUs with >50 sequences), Figure S5: Number of unclassified 16S OTUs at predicted family level obtained per lichen sample, Figure S6: Evolutionary history based on 16S rRNA gene V3-V4 region unclassified sequences of representative operational taxonomic units (OTUs) above *n* = 50, Figure S7: Completeness assignment based on 1706 expected gene content using BUSCO, Figure S8: Taxonomic assignment of shotgun sequences obtained using different databases in MG-RAST, Figure S9: Functional assignment identified at the highest level using EGG NOG, COG, KEGG Orthologs, and SEED subsystems in the MG-RAST server, Figure S10: Hybrids of biosynthetic gene clusters containing both NRPS and PKS biosynthetic genes, Figure S11: Biosynthetic gene clusters containing terpene biosynthetic genes from metagenomes of studied strains and genomes of isolated *Cladonia* fungi, Figure S12: Type I PKS biosynthetic gene clusters, Figure S13: Taxonomic assignment based on Average Amino-acid Identity (AAI) values comparing 129 protein sequences predicted to be involved in the synthesis of PKS type I detected by fungiSMASH, Figure S14: Relative amounts of the lichen SMs identified by ESI-Q-TOF mass spectrometric detection, Figure S15: Examples of chromatograms and spectra indicating the lichen compounds detected in the studied samples, Table S1: Number of sequences assigned to green algae per sample, Table S2: Relative amount of reads from ITS2 amplicon OTU analysis that were classified to species of *Cladonia* and other classifications, Table S3: Summary of the quality assessment of the studied sequences calculated using QUAST using the genome of *Cladonia rangiferina* ATCC 18275 (ASM614605v1) as reference, Table S4: Summary of the quality assessment of the studied sequences calculated using MetaQUAST, Table S5: Sequenced metagenomes generated in this study, Table S6: Published genomes of *Cladonia* included in the bioinformatic analyses, Table S7: Summary of biosynthetic gene cluster types found using bacterial antiSMASH at standalone for metagenomic data (*_anti) and fungal antiSMASH at web (*_fungi), Table S8. Compounds detected in lichen acetone (Ac) or methanol (Me) extracts from samples kept at room temperature or frozen, List 1: Unicellular fungi and green algae sequences added to the UNITE library, List 2: Compounds screened using the software UNIFI scientific information system (Waters).
