# Supplementary material for: Microbial Communities of Cladonia Lichens and Their Biosynthetic Gene Clusters Potentially Encoding Natural Products
Source: Microorganisms. 2021 Jun 22;9(7):1347. doi: 10.3390/microorganisms9071347 (PMC8304397; doi:10.3390/microorganisms9071347)
Supplement: Supplementary file 1 [file microorganisms-09-01347-s001.zip › shishidoetal_supplementary.pdf]

Supplementary material

Microbial Communities of *Cladonia* Lichens and Their Biosynthetic Gene Clusters Potentially Encoding Natural Products

Tânia Keiko Shishido [1,*], Matti Wahlsten [2], Pia Laine [1], Jouko Rikkinen [3,4], Taina Lundell [2] and Petri Auvinen [1]

[1] Institute of Biotechnology, University of Helsinki, P.O.Box 56, 00014 Helsinki, Finland; pia.k.laine@helsinki.fi (P.L.); petri.auvinen@helsinki.fi (P.A.)
[2] Department of Microbiology, Faculty of Agriculture and Forestry, University of Helsinki, P.O.Box 56, 00014 Helsinki, Finland; matti.wahlsten@helsinki.fi (M.W.); taina.lundell@helsinki.fi (T.L.)
[3] Finnish Museum of Natural History, Botany Unit, University of Helsinki, P.O. Box 7, 00014 Helsinki, Finland; jouko.rikkinen@helsinki.fi
[4] Organismal and Evolutionary Biology Research Programme, Faculty of Biological and Environmental Sciences, University of Helsinki, P.O. Box 65, 00014 Helsinki, Finland
* Correspondence: taniakeiko@gmail.com

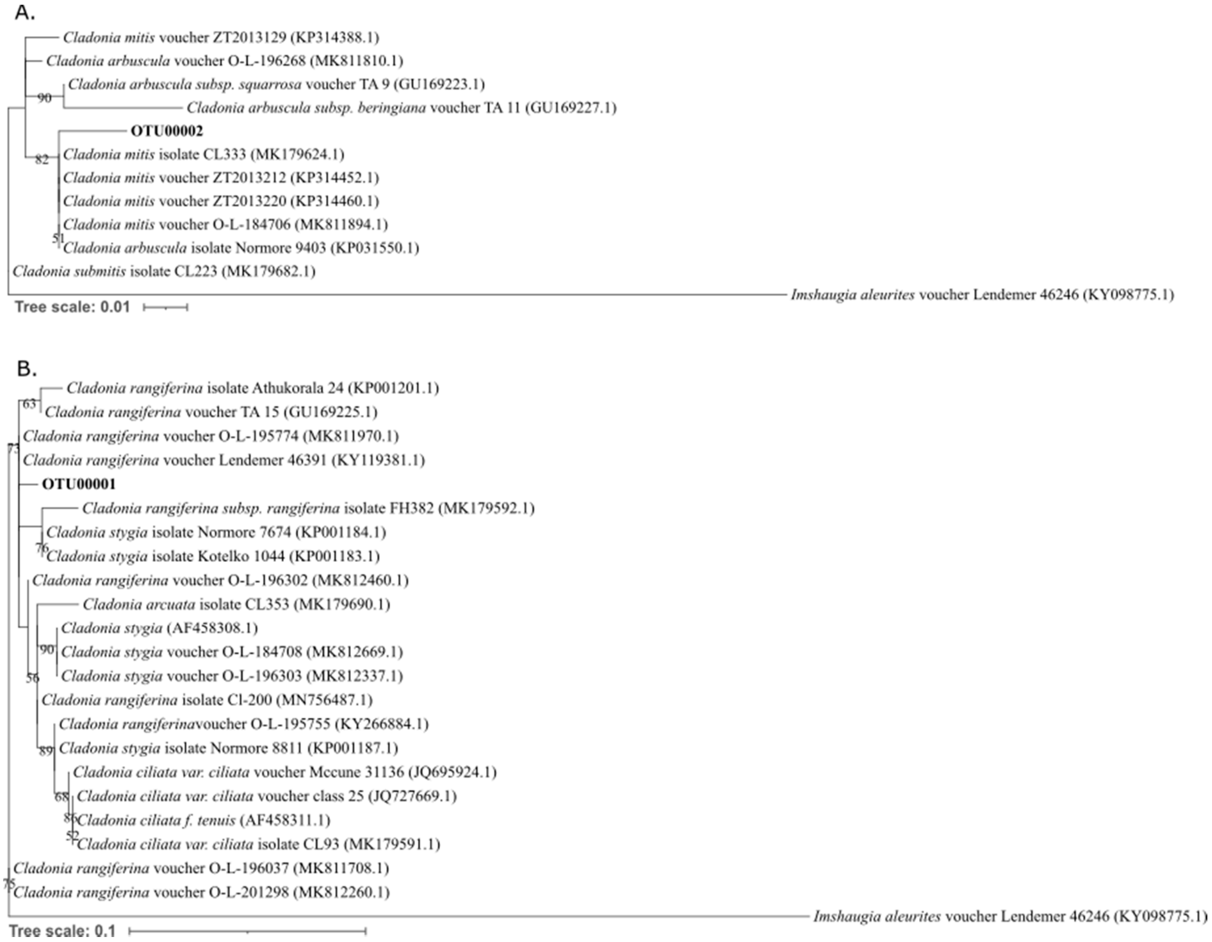

Figure S1. Maximum likelihood phylogenetic tree constructed using representative ITS2 amplicon sequences of two operational taxonomic units OTU 00002 (A) and OTU00001 (B) obtained from mothur analysis. Values above 50 % for the support of each node after 1000 bootstraps are shown.

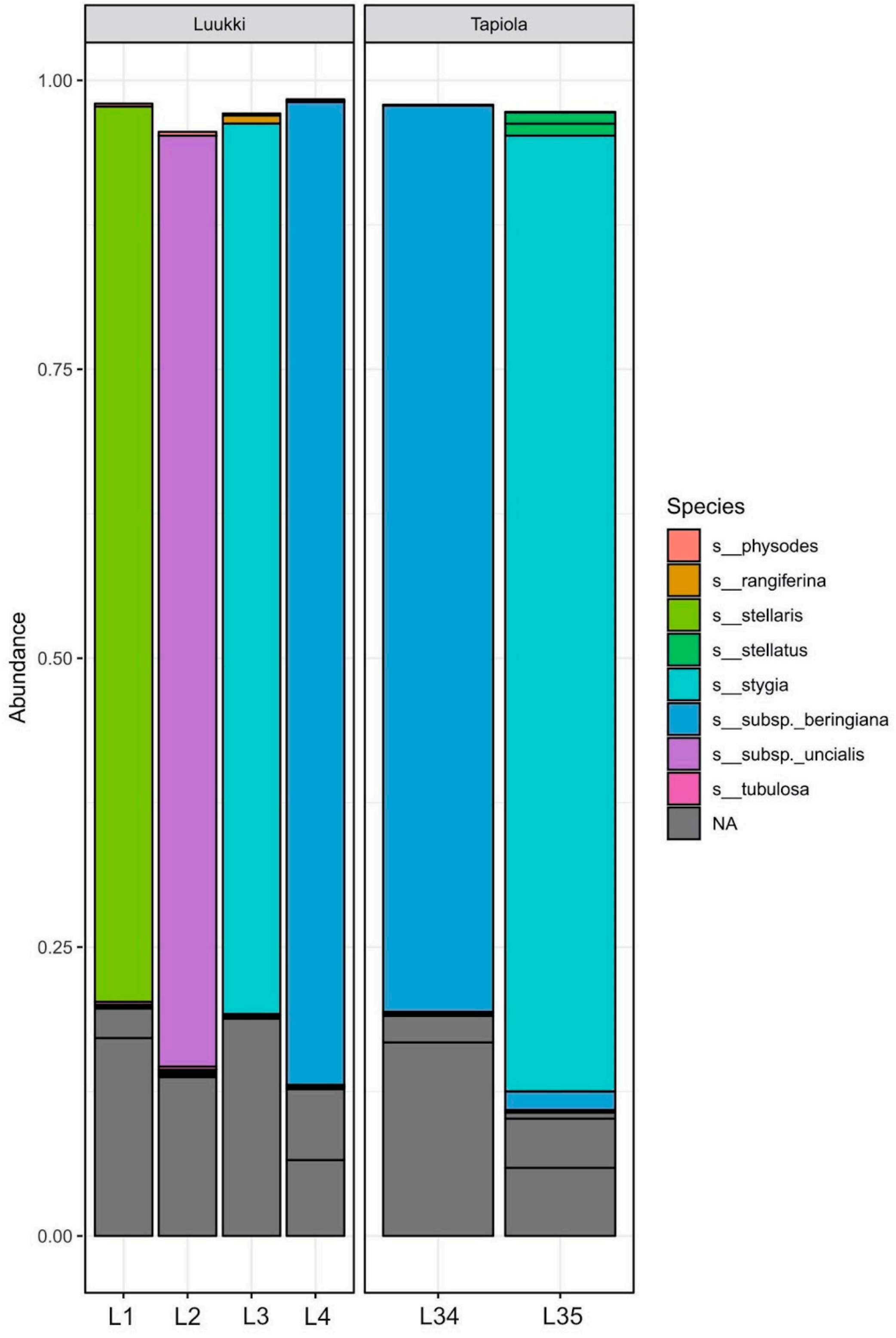

Figure S2. Microbial community at species taxonomic level inferred by DADA2 based on amplicon sequence variants (ASVs) from samples collected from a boreal forest (Luukki) and urban area (Tapiola) sites.

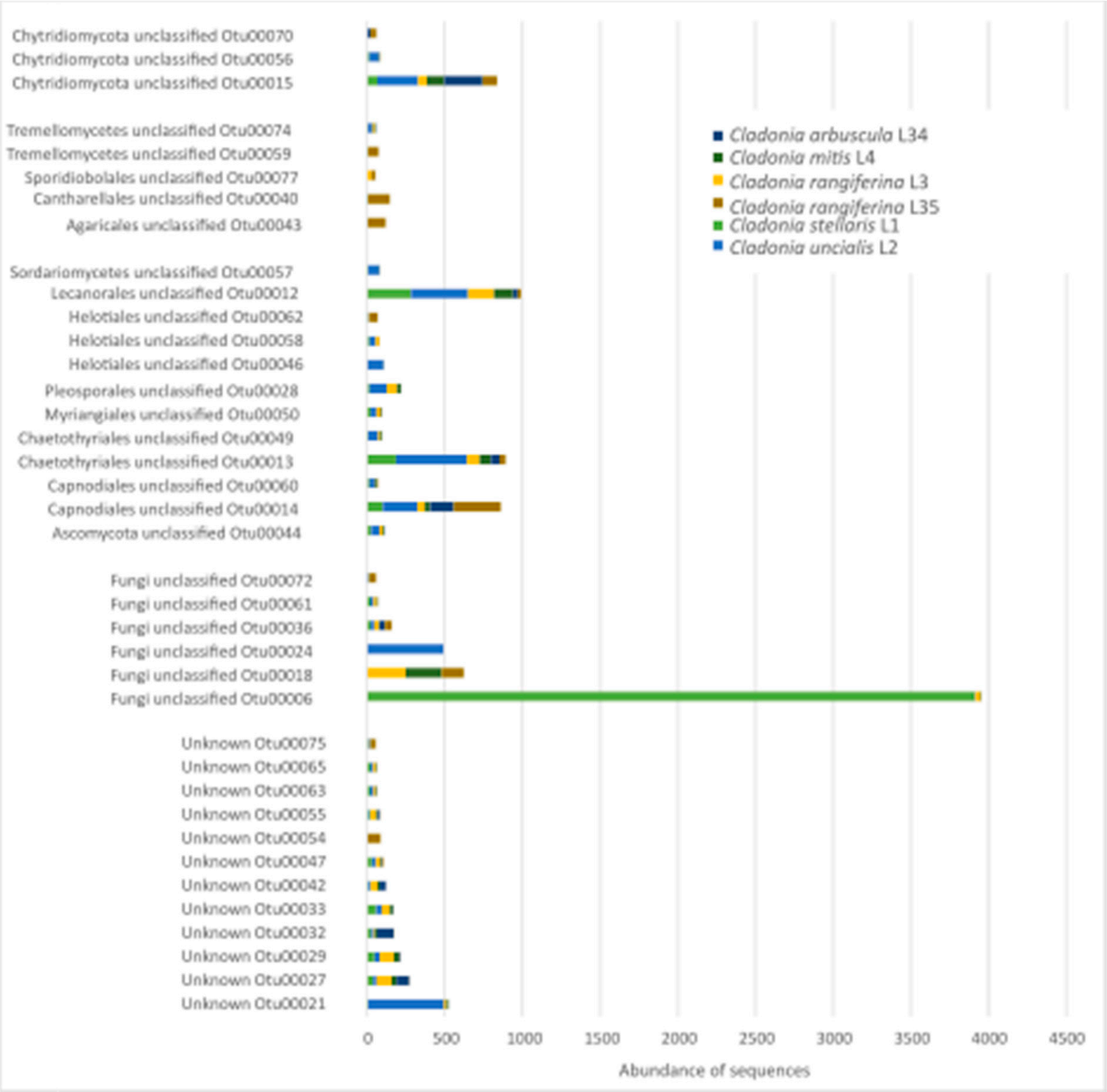

Figure S3. Number of unclassified ITS2 sequences at family taxonomic level (n > 50) per sample.

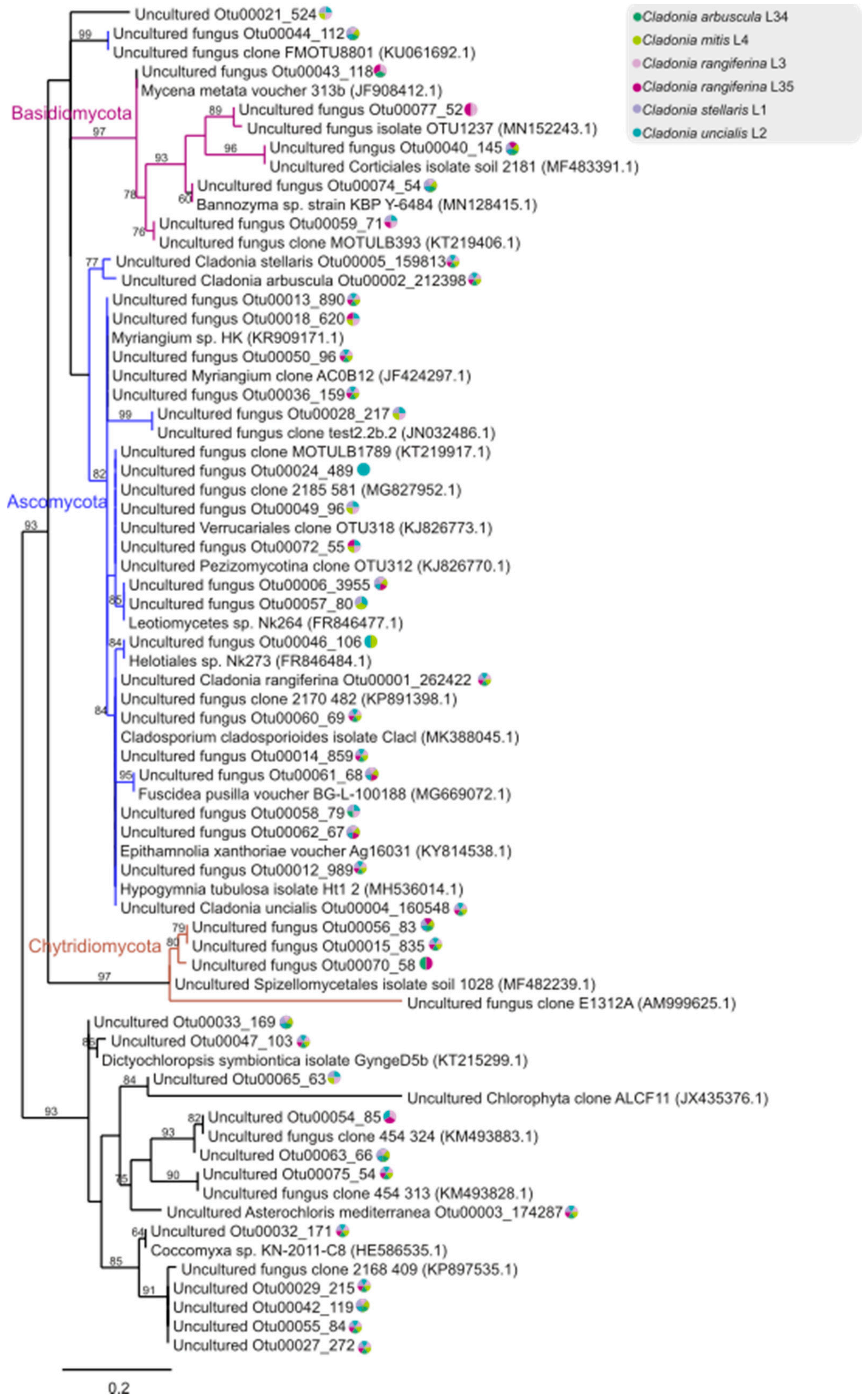

Figure S4. Evolutionary history based on ITS2 unclassified operational taxonomic units (OTUs with above 50 sequences). The legends indicate the samples which contained the OTUs. Otu0006 mentioned in the main text is shown in bold.

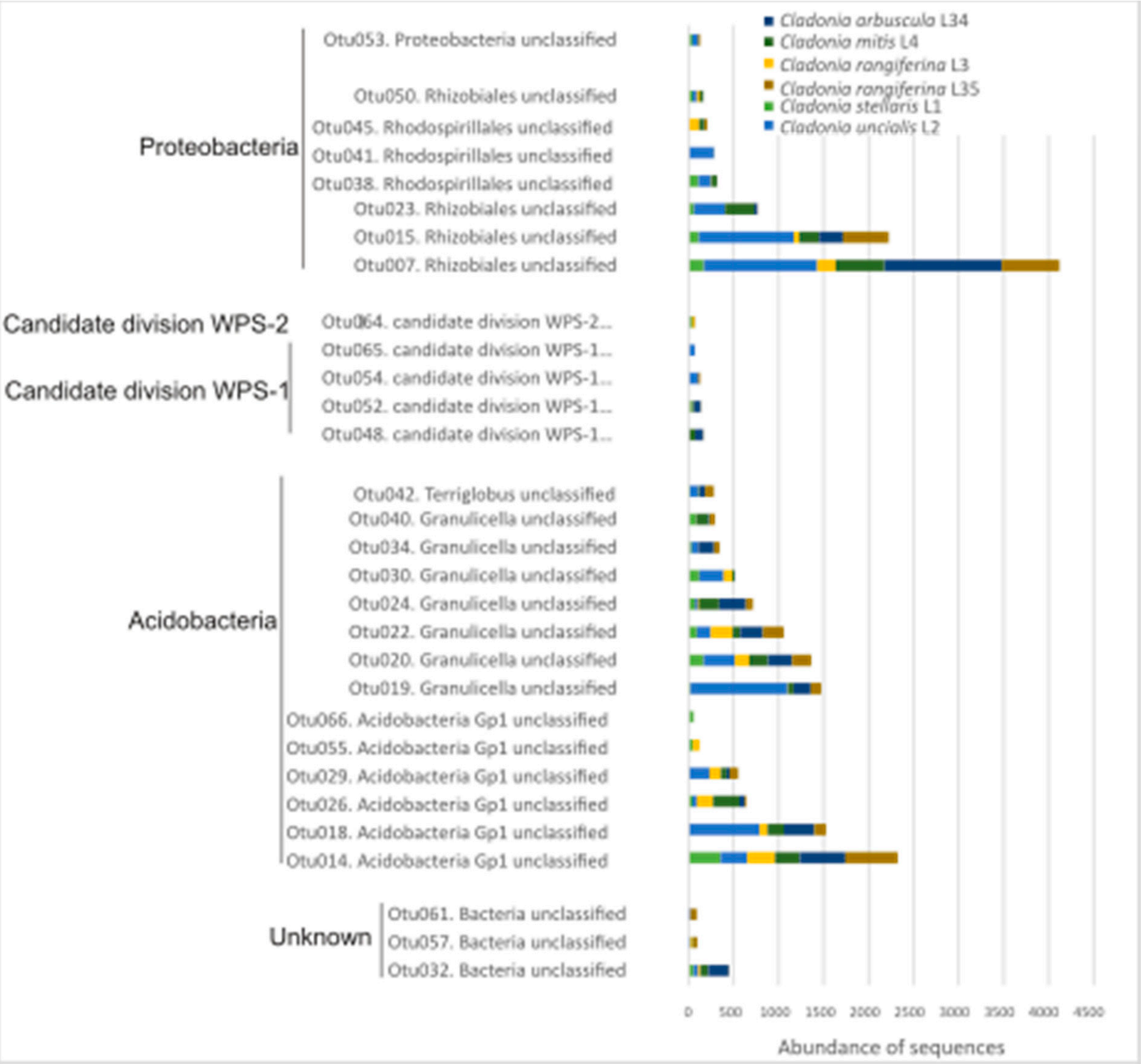

Figure S5. Number of unclassified 16S OTUs at predicted family level obtained per lichen sample.

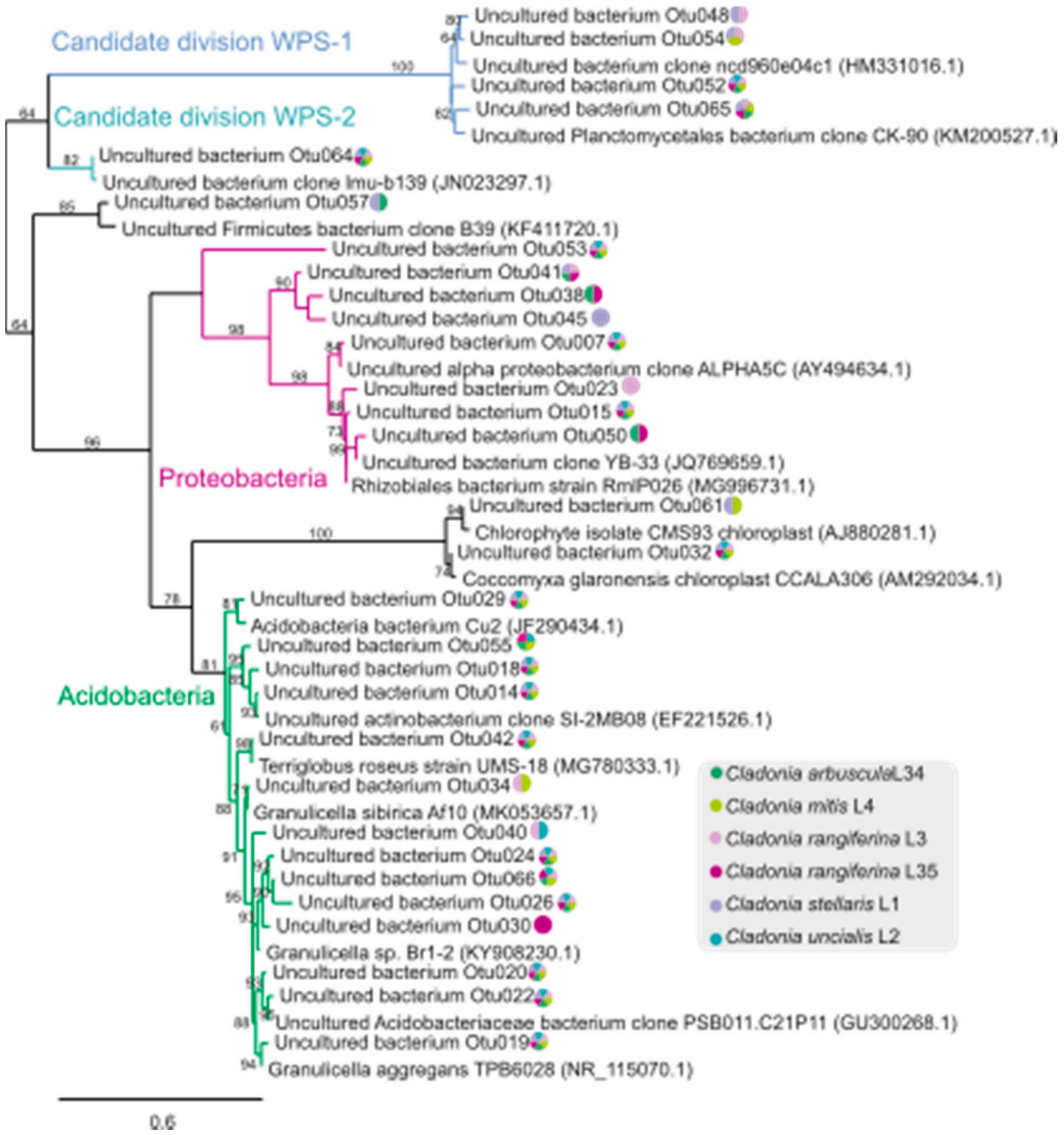

Figure S6. Evolutionary history based on 16S rRNA gene V3-V4 region unclassified sequences of representative operational taxonomic units (OTUs) above n = 50. The legends indicate the lichen samples which contained the OTUs.

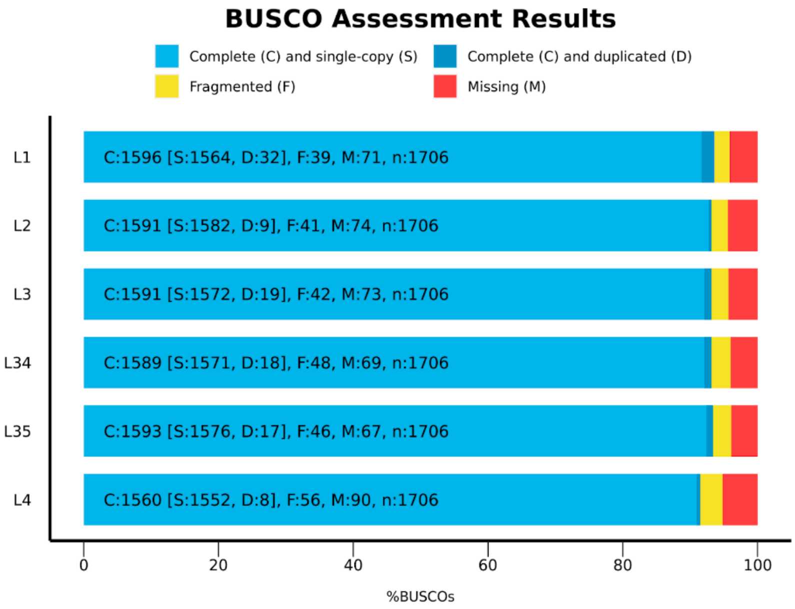

Figure S7. Completeness assignment based on 1706 expected gene content using BUSCO.

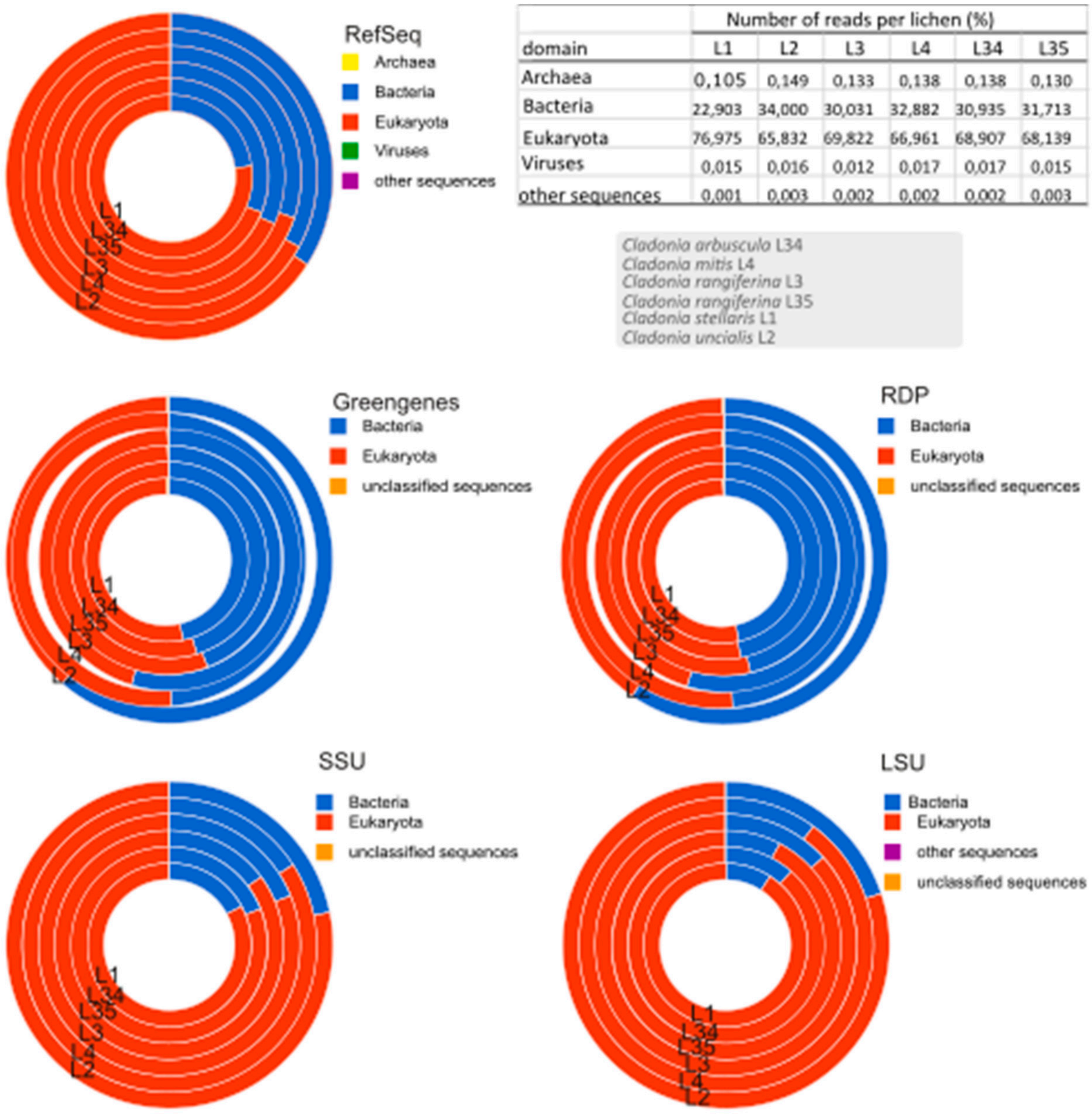

	Number of reads per lichen (%)					
domain	L1	L2	L3	L4	L34	L35
Archaea	0,105	0,149	0,133	0,138	0,138	0,130
Bacteria	22,903	34,000	30,031	32,882	30,935	31,713
Eukaryota	76,975	65,832	69,822	66,961	68,907	68,139
Viruses	0,015	0,016	0,012	0,017	0,017	0,015
other sequences	0,001	0,003	0,002	0,002	0,002	0,003

Figure S8. Taxonomic assignment of shotgun sequences obtained using different databases in MG-RAST. The inserted table indicates the percentage of reads assigned to different taxonomic taxa.

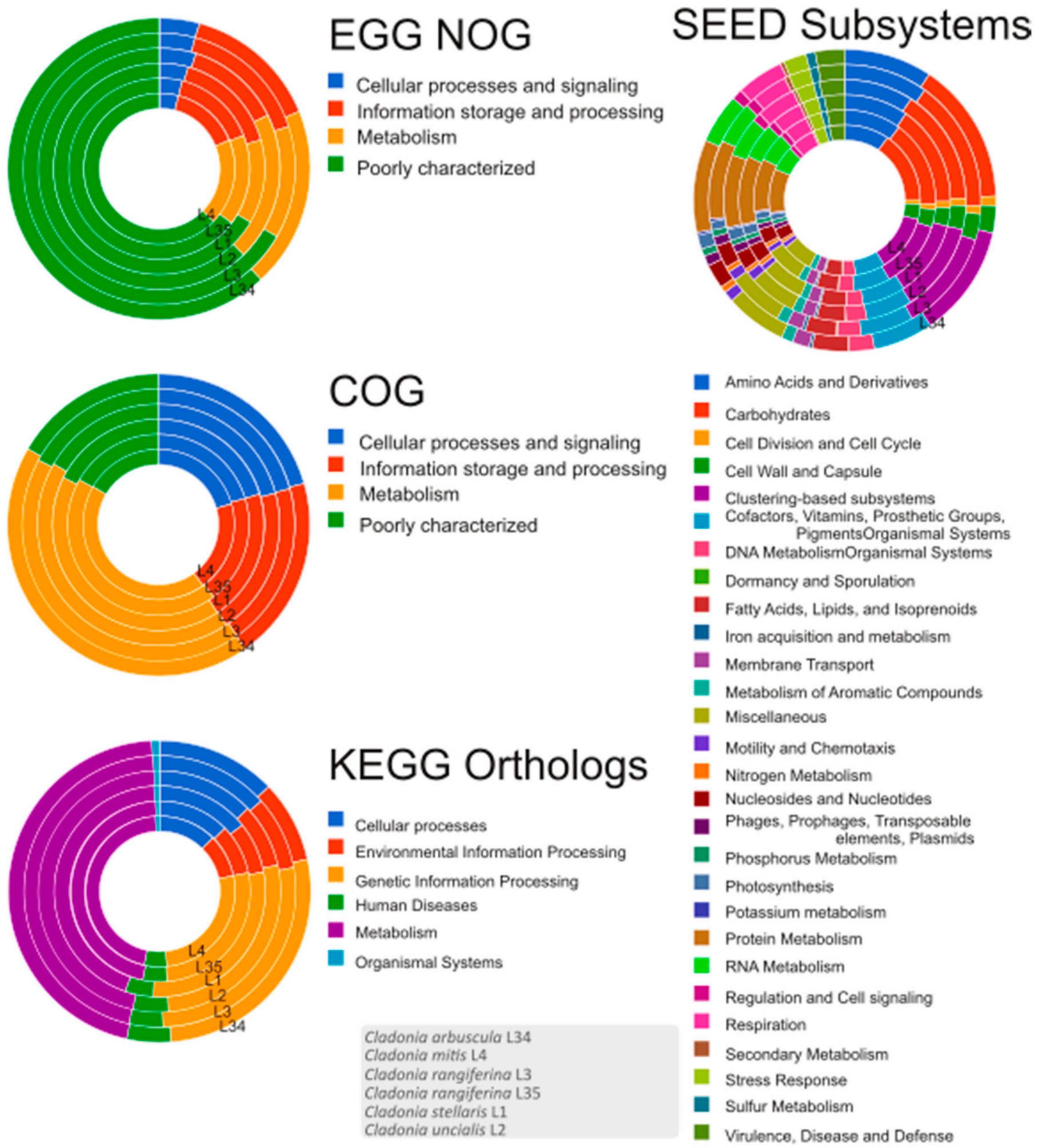

Figure S9. Functional assignment identified at the highest level using EGG NOG, COG, KEGG Orthologs and SEED subsystems in the MG-RAST server. Sequences with assigned function represented 44 to 49% (L34 -44.22%, L35 -44.68%, L1 -46.21%, L2 -45.19%, L3 -49.21%, L4 -43.69%) of the total raw sequences submitted to MG-RAST.

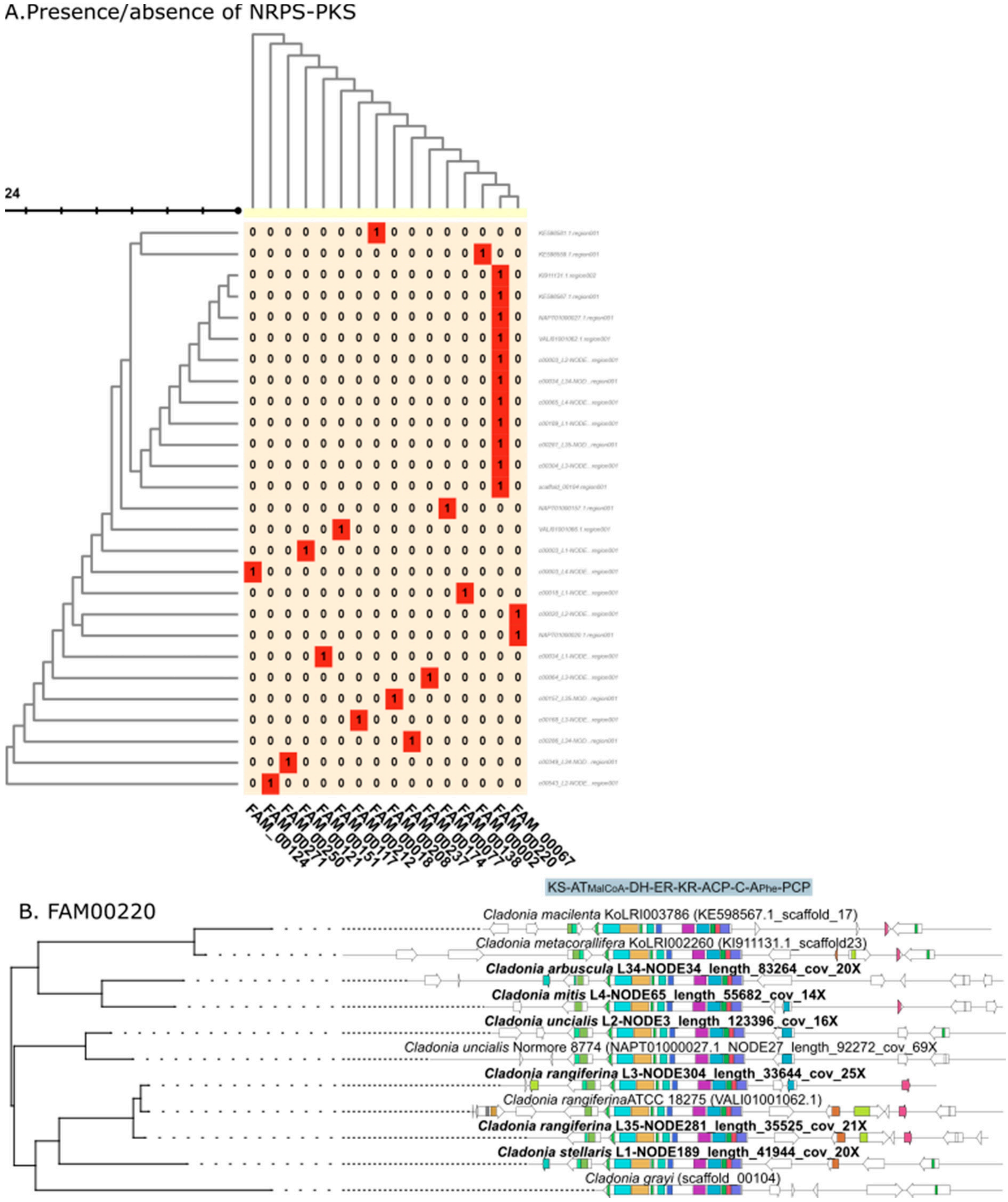

Figure S10. Hybrids of biosynthetic gene clusters containing both NRPS and PKS biosynthetic genes. A. Heatmap based on presence/absence of the 20 largest NRPS-PKS hybrid gene cluster families from lichen metagenomes obtained in this study and in sequenced genomes of *Cladonia* fungi. B. Phylogenetic history of hybrid NRPS and PKS biosynthetic gene cluster (FAM_00220) obtained during BiG-SCAPE/CORASON analysis. All metagenomes (L1-L34, in bold) and *Cladonia* genomes have similar core biosynthetic protein with the same order of domains and same substrate prediction for the adenylation (phenylalanine) and acyltransferase (malonyl CoA) domains.

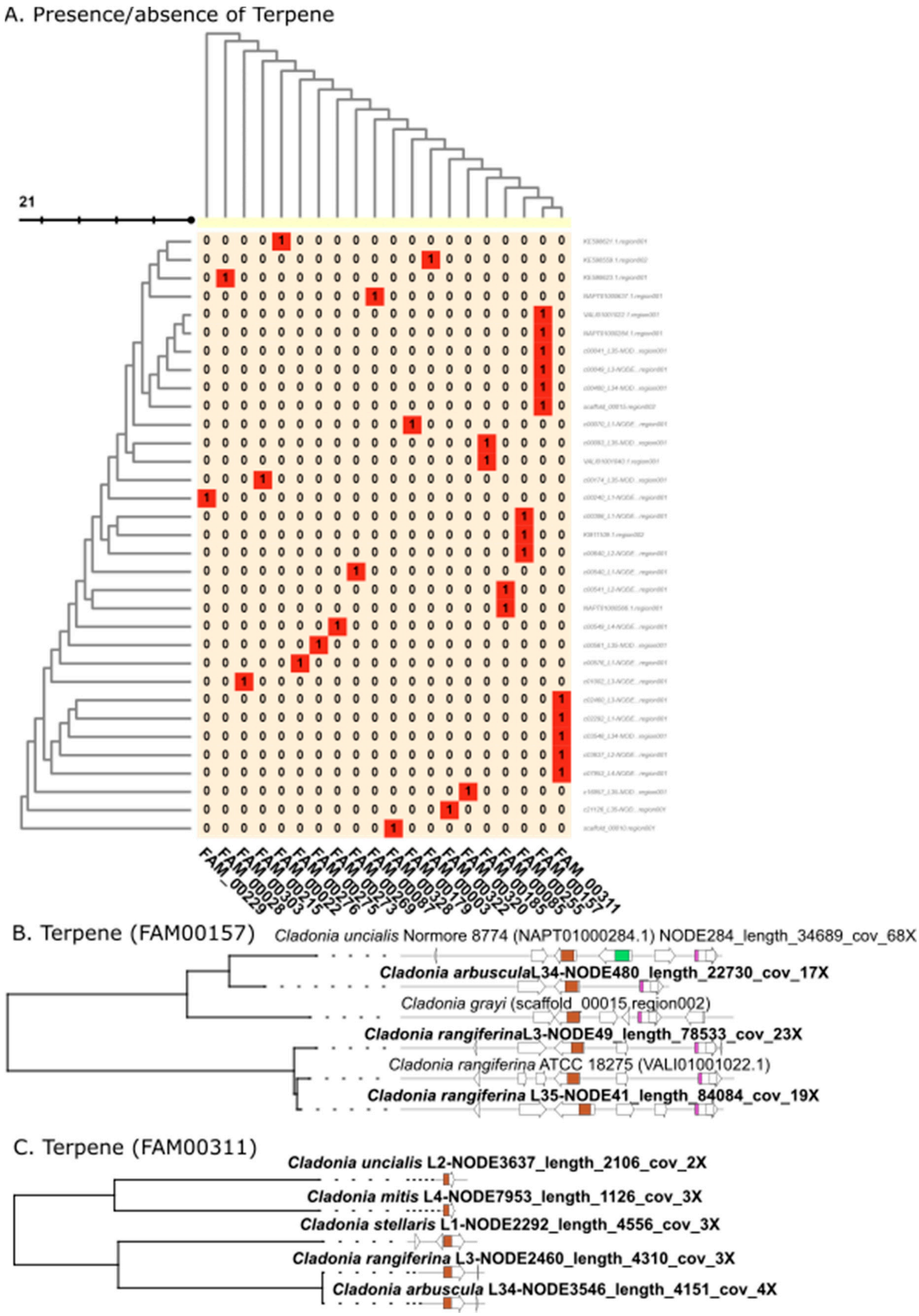

Figure S11. Biosynthetic gene clusters containing terpenes biosynthetic genes from metagenomes of studied strains and genomes of isolated *Cladonia* fungi. A. Heatmap based on presence/absence of the 20 largest Terpene families detected in the lichen metagenomes obtained in this study and in sequenced genomes of *Cladonia* species. Gene clusters families FAM00157 (B) and FAM00311 (C) detected in different *Cladonia* genomes/metagenomes using fungiSMASH and BiG-SCAPE/CORASON. Studied strains (data obtained from metagenomes) are highlighted in bold.

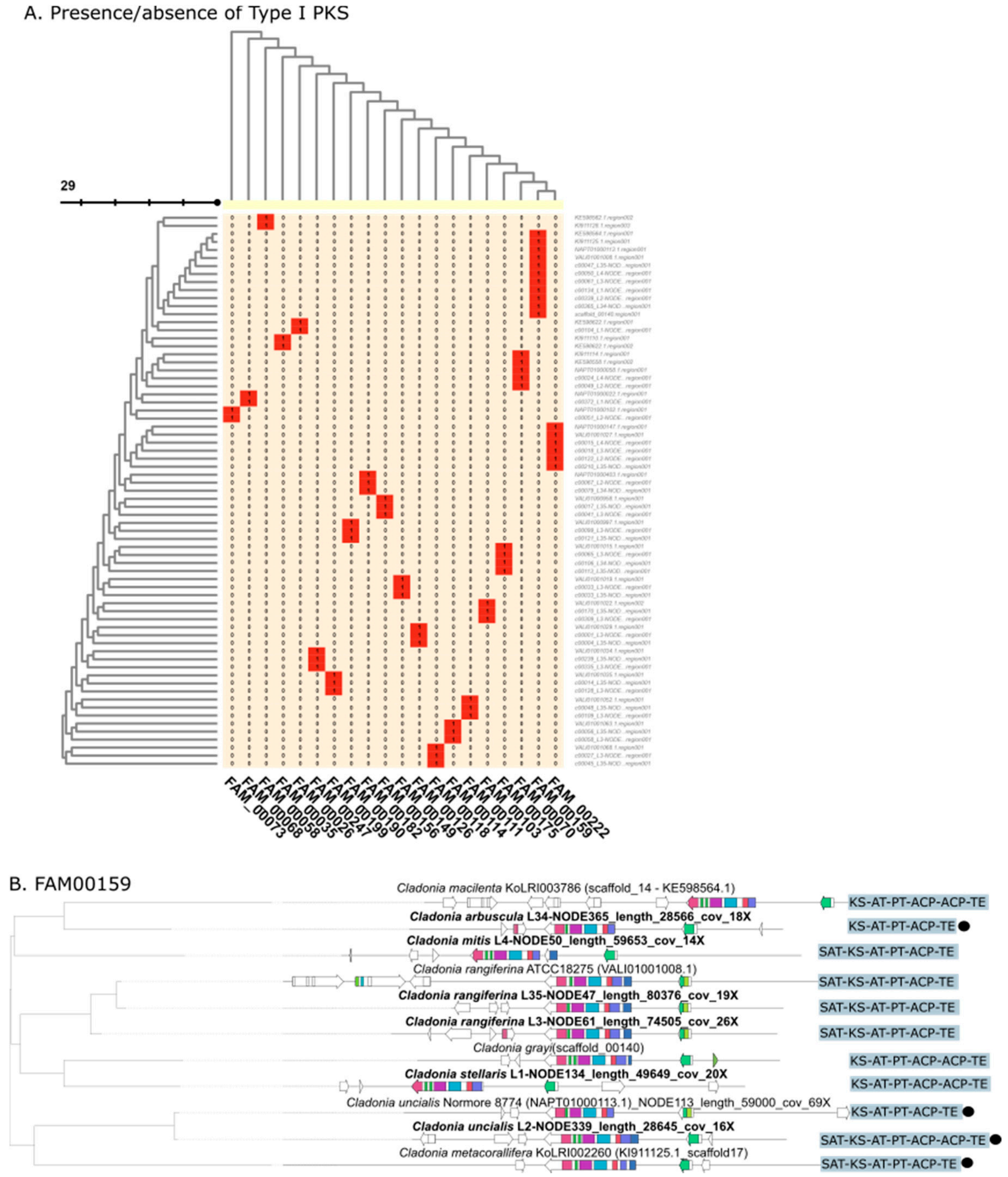

Figure S12. Type I PKS biosynthetic gene clusters. A. Heatmap based on presence/absence of the 20 largest Type I PKS families detected in the lichen metagenomes obtained in this study and in sequenced genomes of *Cladonia* fungi. B. Biosynthetic gene clusters containing PKS type I biosynthetic genes (FAM00159) detected in different *Cladonia* genomes/metagenomes. Studied strains (data obtained from metagenomes) are highlighted in bold. The prediction by fungiSMASH of *Cladonia* biosynthetic genes to be 100% similar to 1,3,6,8-tetrahydroxynaphthalene (MIBiG: BGC0001258) are indicated with a circle. Other samples returned with a similarity of 22% to naphthalene biosynthetic pathway (MIBiG:BGC0001906).

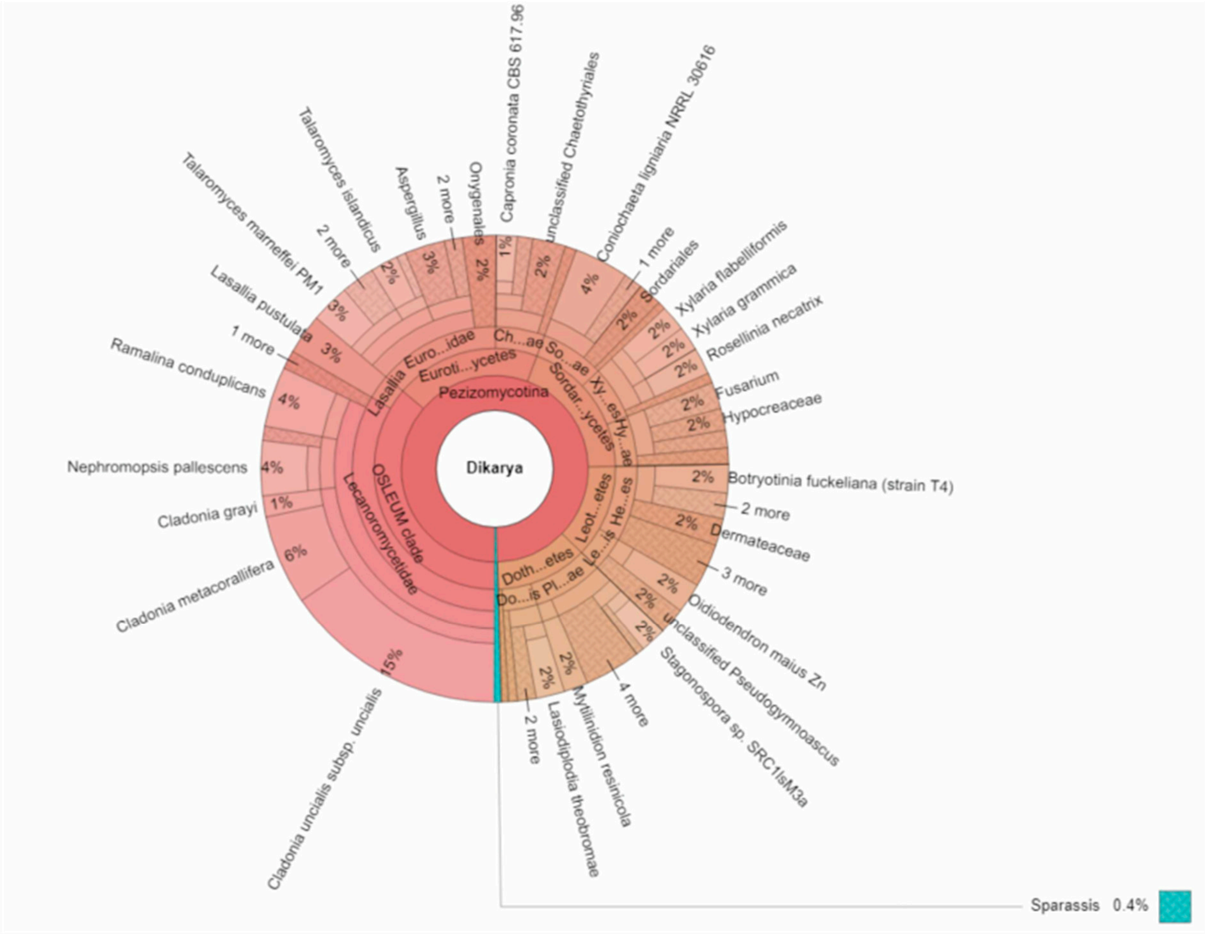

Figure S13. Taxonomic assignment based on Average Amino-acid Identity (AAI) values comparing 129 protein sequences predicted to be involved in the synthesis of PKS type I detected by fungiSMASH.

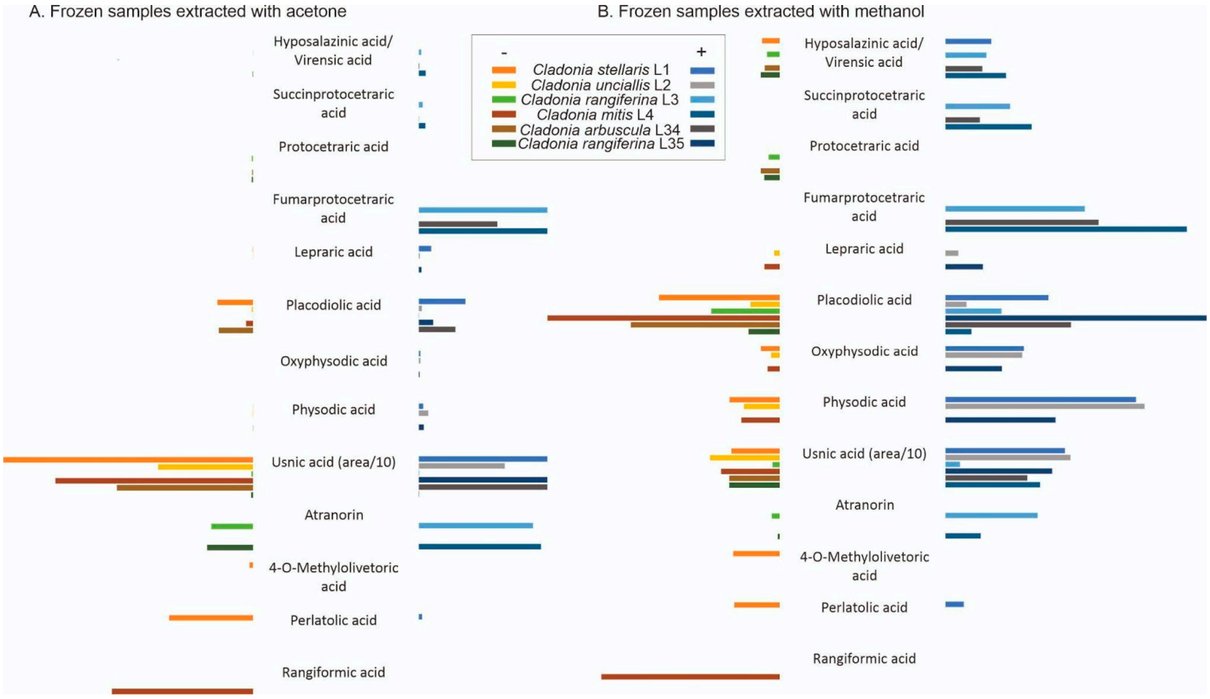

Figure S14. Relative amounts of the lichen secondary metabolites identified by ESI-Q-TOF mass spectrometric detection. Data were analyzed in positive and negative ion modes after compound separation by UPLC from frozen lichen samples extracted using either acetone (A) or methanol (B). Visualized data are derived from Supplementary Table S8.

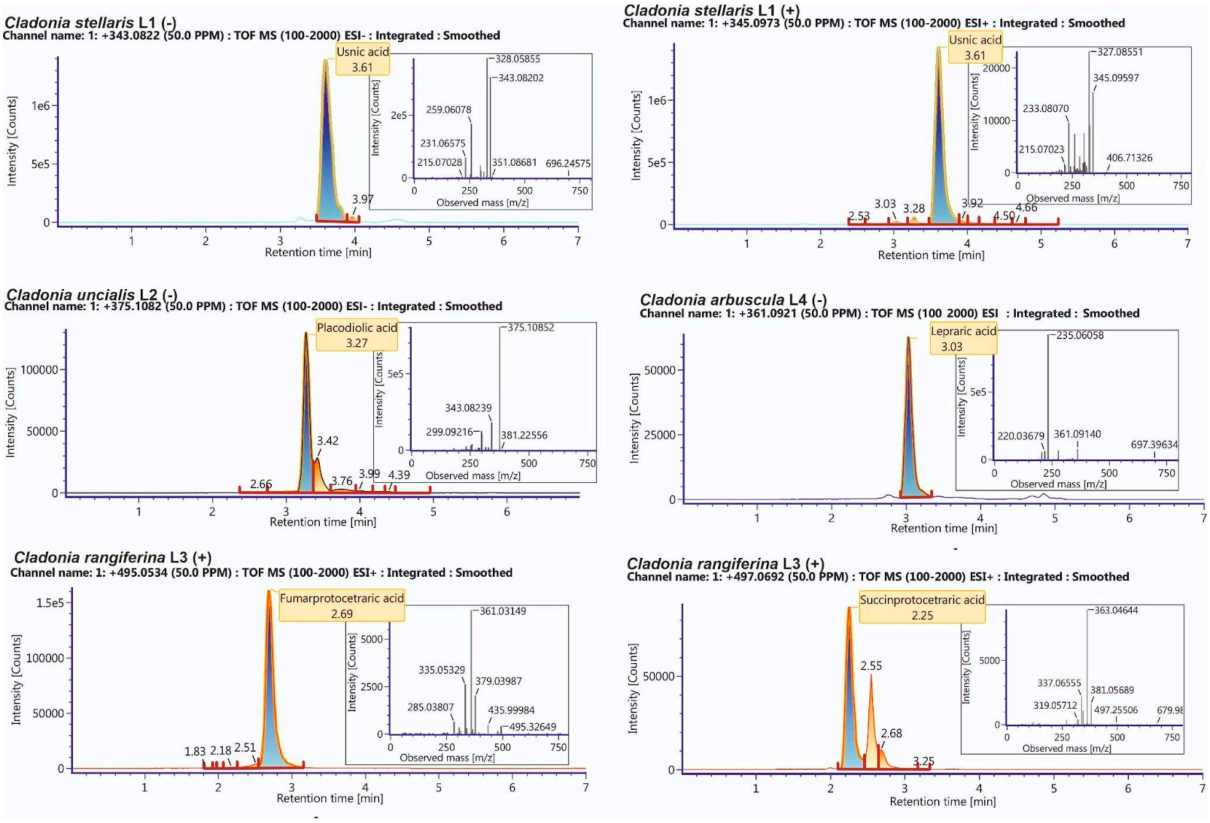

Figure S15. Examples of chromatograms and spectra indicating the lichen compounds detected in the studied samples. Mass spectrometric data was analyzed in positive (+) and negative (-) ion mode.

Table S1. Number of sequences assigned to green algae per sample.

Species	OTU	L1	L2	L3	L4	L34	L35	Total
Asterochloris mediterranea	Otu00003	34797	33283	32291	20075	29518	24318	174282
Trebouxia decolorans	Otu00008	371	113	783	118	43	3	1431
Trebouxia jamesii	Otu00010	32	16	19	21	296	969	1353
Trebouxia jamesii	Otu00011	293	483	172	114	27	23	1112
Trebouxia jamesii	Otu00016	14	7	7	10	171	429	638
Trebouxia jamesii	Otu00017	81	124	44	57	317	10	633
Trebouxia decolorans	Otu00019	275	106	57	133	19	1	591
Trebouxia decolorans	Otu00030	48	51	38	39	31	2	209
Trebouxia jamesii	Otu00031	80	62	38	21	0	0	201
Trebouxia jamesii	Otu00034	35	47	47	28	5	5	167
Trebouxia jamesii	Otu00051	20	13	14	16	28	0	91
Trebouxia jamesii	Otu00064	23	23	12	7	0	0	65
Trebouxia decolorans	Otu00068	27	14	8	8	1	1	59

Table S2. Relative amount of reads from ITS2 amplicon OTU analysis which were classified to species of *Cladonia* and other classifications. Relative abundance of reads above 50 % for each lichen specimen are highlighted in the table.

Relative amount (%)	L1	L2	L3	L4	L34	L35
Cladonia rangiferina subsp rangiferina OTU00001	0,00	0,00	75,07	0,15	0,00	81,23
Cladonia arbuscula OTU00002	0,00	0,00	0,31	82,91	75,17	1,64
Cladonia uncialis subsp biuncialis OTU00004	0,00	79,70	0,00	0,09	0,00	0,00
Cladonia stellaris OTU00005	78,94	0,00	0,00	0,01	0,00	0,00
Unclassified	2,49	1,66	0,89	0,67	0,63	0,67
Other	18,27	17,98	23,36	15,91	23,81	16,00
Rare OTU (<50 reads per OTU)	0,29	0,64	0,36	0,26	0,38	0,45

Table S3. Summary of the quality assessment of the studied sequences calculated using QUAST using the genome of *Cladonia rangiferina* ATCC 18275 (ASM614605v1) as reference.

	new.contigs_L1	new.contigs_L2	new.contigs_L3	new.contigs_L4	new.contigs_L34	new.contigs_L35
# contigs (>= 0 bp)	100526	131864	126771	116310	153379	159796
# contigs (>= 1000 bp)	20102	16513	20835	10331	20597	21226
# contigs (>= 5000 bp)	1968	1289	1892	1459	2534	2192
# contigs (>= 10000 bp)	921	924	890	1014	985	887
# contigs (>= 25000 bp)	413	405	438	390	427	430
# contigs (>= 50000 bp)	130	138	159	87	144	157
Total length (>= 0 bp)	100838840	100203797	111939391	84030123	125533981	127848409
Total length (>= 1000 bp)	71152244	54508614	73472126	42995763	77586544	76471750
Total length (>= 5000 bp)	33916435	30312139	35307578	29837639	39581743	37290339
Total length (>= 10000 bp)	27102029	27685055	28803504	26549490	29341120	28766078
Total length (>= 25000 bp)	19023593	19147181	21458724	16345530	20380765	21469387
Total length (>= 50000 bp)	9033646	9960752	11703313	5846122	10439480	11771241
# contigs	35847	45212	40205	34739	43712	48298
Largest contig	138857	154269	158143	147081	176544	184648
Total length	82094879	74420864	86876711	59735367	93106593	94724226
Reference length	35673945	35673945	35673945	35673945	35673945	35673945
GC (%)	54.28	53.64	54.50	52.87	54.28	54.27
Reference GC (%)	45.44	45.44	45.44	45.44	45.44	45.44
N50	3501	2059	3362	4902	3774	3275
NG50	27372	27729	32474	22619	30651	33800
N75	1599	955	1460	920	1482	1293
NG75	10527	11564	14190	9725	13907	13440
L50	3705	3812	3918	1466	4147	4729
LG50	368	355	312	453	335	304
L75	12538	17852	13956	12215	14207	16434
LG75	888	838	718	1035	763	716
# N's per 100 kbp	0.00	0.00	0.00	0.00	0.00	0.00
NGA50	-	-	-	-	-	-

All statistics are based on contigs of size >= 500 bp, unless otherwise noted (e.g., "# contigs (>= 0 bp)" and "Total length (>= 0 bp)" include all contigs).

Table S4. Summary of the quality assessment of the studied sequences calculated using MetaQUAST. The combined genome reference consisted of: *Acetobacter tropicalis*, *Acidiphilium multivorum* AIU301, *Acidocella* sp. MX AZ02, *Algisphaera agarilytica*, *Anabaena variabilis* ATCC 29413, *Aquisphaera giovannonii*, *Bradyrhizobium japonicum*, *Brevundimonas nasdae*, *Caulobacter vibrioides*, *Chlorella sorokiniana*, *Chthoniobacter flavus*, *Gluconacetobacter diazotrophicus* PA1 5, *Gluconacetobacter liquefaciens*, *Gluconacetobacter takamatsuzukensis*, *Granulibacter bethesdensis* CGDNIH4, *Kamptonema formosum* PCC 6407, *Komagataeibacter kakiaceti*, *Kozakia baliensis*, *Methylobacterium soli*, *Methylocystis heyeri*, *Phormidium* sp. CENA135, *Rhodoblastus sphagnicola*, *Rhodopila globiformis*, *Rhodovastum atsumiense*, *Singulisphaera acidiphila*, *Talaromyces rugulosus*.

	new.contigs_L1	new.contigs_L2	new.contigs_L3	new.contigs_L4	new.contigs_L34	new.contigs_L35
# contigs (>= 0 bp)	100526	131864	126771	116310	153379	159796
# contigs (>= 1000 bp)	20102	16513	20835	10331	20597	21226
# contigs (>= 5000 bp)	1968	1289	1892	1459	2534	2192
# contigs (>= 10000 bp)	921	924	890	1014	985	887
# contigs (>= 25000 bp)	413	405	438	390	427	430
# contigs (>= 50000 bp)	130	138	159	87	144	157
Total length (>= 0 bp)	100838840	100203797	111939391	84030123	125533981	127848409
Total length (>= 1000 bp)	71152244	54508614	73472126	42995763	77586544	76471750
Total length (>= 5000 bp)	33916435	30312139	35307578	29837639	39581743	37290339
Total length (>= 10000 bp)	27102029	27685055	28803504	26549490	29341120	28766078
Total length (>= 25000 bp)	19023593	19147181	21458724	16345530	20380765	21469387
Total length (>= 50000 bp)	9033646	9960752	11703313	5846122	10439480	11771241
# contigs	35847	45212	40205	34739	43712	48298
Largest contig	138857	154269	158143	147081	176544	184648
Total length	82094879	74420864	86876711	59735367	93106593	94724226
Reference length	227932695	227932695	227932695	227932695	227932695	227932695
N50	3501	2059	3362	4902	3774	3275
N75	1599	955	1460	920	1482	1293
L50	3705	3812	3918	1466	4147	4729
L75	12538	17852	13956	12215	14207	16434
# N's per 100 kbp	0.00	0.00	0.00	0.00	0.00	0.00

All statistics are based on contigs of size >= 500 bp, unless otherwise noted
(e.g., "# contigs (>= 0 bp)" and "Total length (>= 0 bp)" include all contigs).

Table S5. Sequenced metagenomes generated in this study. *Raw sequences are available under accession number PRJEB34718. **Compounds described in Figure 4 and Supplementary Figure S14.

Species and strain	Sequencer	Assembler	Assembled metagenome (Mbp)	Genome coverage	Number of contigs	Accession number	Metabolites
Metagenomes							
Cladonia arbuscula L34	Illumina NextSeq500	SPAdes 3.12.0	93	-	19649	*	**
Cladonia mitis L4	Illumina NextSeq500	SPAdes 3.12.0	59	-	11760	*	**
Cladonia rangiferina L3	Illumina NextSeq500	SPAdes 3.12.0	86	-	20256	*	**
Cladonia rangiferina L35	Illumina NextSeq500	SPAdes 3.12.0	94	-	20592	*	**
Cladonia stellaris L1	Illumina NextSeq500	SPAdes 3.12.0	82	-	19362	*	**
Cladonia uncialis L2	Illumina NextSeq500	SPAdes 3.12.0	74	-	17633	*	**

Table S6. Published genomes of *Cladonia* included in the bioinformatic analyses.

Genomes	Sequencer	Assembler	Assembled genome (Mbp)	Genome coverage	Number of contigs	Accession number	Metabolites	Reference
Cladonia grayi	454 and Solexa gDNA	Velvet and Newbler	34.62	15x	414	Cladonia grayi Cgr/DA2myc/ss v1.0	Grayanic acid, fumarprotocetraric acid	Armaleo et al. 2019, Calchera et al. 2019
Cladonia macilenta KoLRI003786	Illumina HiSeq2000	SOAPdenovo	37.11	540x	240	AUPP00000000	Thamnolic, barbatic, didymic, squamatic, usnic and rhodocladonic acids	Park et al. 2013, Calchera et al. 2019
Cladonia metacorallifera KoLRI002260*	Illumina HiSeq2000	Allpaths-LG	36.68	1023x	30	AXCT00000000	Usnic, didymic, squamatic and rhodocladonic acids	Park et al. 2014, Calchera et al. 2019
Cladonia rangiferina ATCC 18275	Illumina MiSeq	ABySS v. AUG-2016	35.6	1,567	8	ASM614605v1	-	ASM614605v1
Cladonia uncialis Normore8774	Illumina MiSeq	SPAdes v. 3.11	32.85	43x	2124	ASM292778v1	Usnic and squamatic acids	Abdel-Hameed et al. 2016, Calchera et al. 2019

Table S7. Summary of biosynthetic gene cluster types found using bacterial antiSMASH at standalone for metagenomic data (*_anti) and fungal antiSMASH at web (*_fungi).

Sample	T1PKS	T3PKS	Terpene	NRPS	NRPS-T1PKS	T3PKS-T1PKS	Indole	hybrid/T1PKS - indole/terpene/other	Siderophore	Other	Arylpolyene/lassopeptide/bacteriocin/thiopeptide/betalactone	Total
**Metagenomes**												
***Cladonia arbuscula* L34_fungi**	20	2	3	7	3	0	2	1	0	0	0	38
***Cladonia arbuscula* L34_anti**	16	3	5	12	4	0	3	1	0	1	1	46
***Cladonia mitis* L4_fungi**	28	2	2	5	2	0	0	1	1	0	0	41
***Cladonia mitis* L4_anti**	20	4	5	16	2	0	1	0	1	1	0	50
***Cladonia rangiferina* L3_fungi**	19	1	4	6	3	1	0	0	0	0	0	34
***Cladonia rangiferina* L3_anti**	18	4	6	14	1	0	0	1	1	1	1	47
***Cladonia rangiferina* L35_fungi**	17	1	6	4	2	1	0	1	0	0	0	32
***Cladonia rangiferina* L35_anti**	17	3	9	14	1	0	0	0	1	1	1	47
***Cladonia stellaris* L1_fungi**	12	0	6	5	4	1	0	2	0	1	0	31
***Cladonia stellaris* L1_anti**	16	4	6	12	3	0	1	1	0	2	1	46
***Cladonia uncialis* L2_fungi**	15	0	4	5	3	1	0	0	0	0	0	28
***Cladonia uncialis* L2_anti**	16	2	7	14	1	0	1	0	0	0	1	42
**Genomes**												
***Cladonia grayi*_fungi**	12	0	2	9	1	0	0	0	0	1	0	25
***Cladonia grayi*_anti**	8	3	2	11	1	0	1	1	0	1	0	28

***Cladonia macilenta* KoLRI003786_fungi**	18	0	3	6	3	1	0	0	0	0	0	31
***Cladonia macilenta* KoLRI003786_anti**	17	2	4	12	3	0	0	0	0	1	0	39
***Cladonia metacoral-lifera* KoLRI002260_fungi**	19	0	5	8	1	1	0	1	0	1	0	36
***Cladonia metacoral-lifera* KoLRI002260_anti**	15	2	5	12	2	0	0	1	0	0	0	37
***Cladonia rangifer-ina* ATCC 18275 _fungi**	18	0	2	6	2	1	0	1	0	0	0	30
***Cladonia rangifer-ina* ATCC 18275 _anti**	16	3	2	15	1	0	0	2	1	1	1	42
***Cladonia uncialis* Normore8774_fungi**	14	0	3	2	3	1	0	0	0	0	0	23
***Cladonia uncialis* Normore8774_anti**	18	2	4	11	1	0	1	0	0	1	0	38

Table S8. Compounds detected in lichen acetone (Ac) or methanol (Me) extracts from samples kept at room temperature or frozen, List 1: Unicellular fungi and green algae sequences added to the UNITE library, List 2: Compounds screened using the software UNIFI scientific information system (Waters).

List 1. Unicellular fungi and green algae sequences added to the UNITE library.

KU948729
k__Fungi;p__Basidiomycota;c__Cystobasidiomycetes;o__Cyphobasidiales;f__Cyphobasidiaceae;g__Uncultured_Cyphobasidiales;s__Uncultured_Cyphobasidiales;
KU948734
k__Fungi;p__Basidiomycota;c__Cystobasidiomycetes;o__Cyphobasidiales;f__Cyphobasidiaceae;g__Uncultured_Cyphobasidiales;s__Uncultured_Cyphobasidiales;
KU948735
k__Fungi;p__Basidiomycota;c__Cystobasidiomycetes;o__Uncultured_Cystobasidiomycetes;f__Uncultured_Cystobasidiomycetes;g__Uncultured_Cystobasidiomycetes;s__Uncultured_Cystobasidiomycetes;
KU948736
k__Fungi;p__Basidiomycota;c__Cystobasidiomycetes;o__Cyphobasidiales;f__Cyphobasidiaceae;g__Uncultured_Cyphobasidiales;s__Uncultured_Cyphobasidiales;
KU948741
k__Fungi;p__Basidiomycota;c__Cystobasidiomycetes;o__Uncultured_Cystobasidiomycetes;f__Uncultured_Cystobasidiomycetes;g__Uncultured_Cystobasidiomycetes;s__Uncultured_Cystobasidiomycetes;
KU948742
k__Fungi;p__Basidiomycota;c__Cystobasidiomycetes;o__Cyphobasidiales;f__Cyphobasidiaceae;g__Uncultured_Cyphobasidiales;s__Uncultured_Cyphobasidiales;
KU948743
k__Fungi;p__Basidiomycota;c__Cystobasidiomycetes;o__Cyphobasidiales;f__Cyphobasidiaceae;g__Uncultured_Cyphobasidiales;s__Uncultured_Cyphobasidiales;
KU948744
k__Fungi;p__Basidiomycota;c__Cystobasidiomycetes;o__Cyphobasidiales;f__Cyphobasidiaceae;g__Uncultured_Cyphobasidiales;s__Uncultured_Cyphobasidiales;
KU948745 k__Fungi;p__Basidiomycota;c__Cystobasidiomycetes;o__Cyphobasidiales;f__Cyphobasidiaceae;g__Uncultured_Cyphobasidiales;s__Uncultured_Cyphobasidiales;
KU948746
k__Fungi;p__Basidiomycota;c__Cystobasidiomycetes;o__Uncultured_Cystobasidiomycetes;f__Uncultured_Cystobasidiomycetes;g__Uncultured_Cystobasidiomycetes;s__Uncultured_Cystobasidiomycetes;
KU948747
k__Fungi;p__Basidiomycota;c__Cystobasidiomycetes;o__Uncultured_Cystobasidiomycetes;f__Uncultured_Cystobasidiomycetes;g__Uncultured_Cystobasidiomycetes;s__Uncultured_Cystobasidiomycetes;
KU948751
k__Fungi;p__Basidiomycota;c__Cystobasidiomycetes;o__Cyphobasidiales;f__Cyphobasidiaceae;g__Uncultured_Cyphobasidiales;s__Uncultured_Cyphobasidiales;
KU948753
k__Fungi;p__Basidiomycota;c__Cystobasidiomycetes;o__Cyphobasidiales;f__Cyphobasidiaceae;g__Uncultured_Cyphobasidiales;s__Uncultured_Cyphobasidiales;
KU948755
k__Fungi;p__Basidiomycota;c__Cystobasidiomycetes;o__Cyphobasidiales;f__Cyphobasidiaceae;g__Uncultured_Cyphobasidiales;s__Uncultured_Cyphobasidiales;
KU948756
k__Fungi;p__Basidiomycota;c__Cystobasidiomycetes;o__Cyphobasidiales;f__Cyphobasidiaceae;g__Uncultured_Cyphobasidiales;s__Uncultured_Cyphobasidiales;
KU948760
k__Fungi;p__Basidiomycota;c__Cystobasidiomycetes;o__Cyphobasidiales;f__Cyphobasidiaceae;g__Uncultured_Cyphobasidiales;s__Uncultured_Cyphobasidiales;
KU948763
k__Fungi;p__Basidiomycota;c__Cystobasidiomycetes;o__Cyphobasidiales;f__Cyphobasidiaceae;g__Uncultured_Cyphobasidiales;s__Uncultured_Cyphobasidiales;
KU948764
k__Fungi;p__Basidiomycota;c__Cystobasidiomycetes;o__Cyphobasidiales;f__Cyphobasidiaceae;g__Uncultured_Cyphobasidiales;s__Uncultured_Cyphobasidiales;
KU948765

k__Fungi;p__Basidiomycota;c__Cystobasidiomycetes;o__Uncultured_Cystobasidiomycetes;f__Uncultured_Cystobasidiomycetes;g__Uncultured_Cystobasidiomycetes;s__Uncultured_Cystobasidiomycetes;
KU948768
k__Fungi;p__Basidiomycota;c__Cystobasidiomycetes;o__Cyphobasidiales;f__Cyphobasidiaceae;g__Uncultured_Cyphobasidiales;s__Uncultured_Cyphobasidiales;
KU948772
k__Fungi;p__Basidiomycota;c__Cystobasidiomycetes;o__Cyphobasidiales;f__Cyphobasidiaceae;g__Uncultured_Cyphobasidiales;s__Uncultured_Cyphobasidiales;
KU948774
k__Fungi;p__Basidiomycota;c__Cystobasidiomycetes;o__Cyphobasidiales;f__Cyphobasidiaceae;g__Uncultured_Cyphobasidiales;s__Uncultured_Cyphobasidiales;
KU948775
k__Fungi;p__Basidiomycota;c__Cystobasidiomycetes;o__Cyphobasidiales;f__Cyphobasidiaceae;g__Uncultured_Cyphobasidiales;s__Uncultured_Cyphobasidiales;
KU948776
k__Fungi;p__Basidiomycota;c__Cystobasidiomycetes;o__Cyphobasidiales;f__Cyphobasidiaceae;g__Uncultured_Cyphobasidiales;s__Uncultured_Cyphobasidiales;
KU948778
k__Fungi;p__Basidiomycota;c__Cystobasidiomycetes;o__Cyphobasidiales;f__Cyphobasidiaceae;g__Uncultured_Cyphobasidiales;s__Uncultured_Cyphobasidiales;
KU948779
k__Fungi;p__Basidiomycota;c__Cystobasidiomycetes;o__Uncultured_Cystobasidiomycetes;f__Uncultured_Cystobasidiomycetes;g__Uncultured_Cystobasidiomycetes;s__Uncultured_Cystobasidiomycetes;
KU948780
k__Fungi;p__Basidiomycota;c__Cystobasidiomycetes;o__Cyphobasidiales;f__Cyphobasidiaceae;g__Uncultured_Cyphobasidiales;s__Uncultured_Cyphobasidiales;
KU948784
k__Fungi;p__Basidiomycota;c__Cystobasidiomycetes;o__Cyphobasidiales;f__Cyphobasidiaceae;g__Uncultured_Cyphobasidiales;s__Uncultured_Cyphobasidiales;
KU948785
k__Fungi;p__Basidiomycota;c__Cystobasidiomycetes;o__Cyphobasidiales;f__Cyphobasidiaceae;g__Uncultured_Cyphobasidiales;s__Uncultured_Cyphobasidiales;
AB627405
k__Plantae;p__Chlorophyta;c__Trebouxiophyceae;o__Trebouxiales;f__Trebouxiaceae;g__Trebouxia;s__Trebouxia_corticola;
AB627375
k__Plantae;p__Chlorophyta;c__Trebouxiophyceae;o__Trebouxiales;f__Trebouxiaceae;g__Trebouxia;s__Trebouxia_corticola;
JF831923
k__Plantae;p__Chlorophyta;c__Trebouxiophyceae;o__Trebouxiales;f__Trebouxiaceae;g__Trebouxia;s__Trebouxia_decolorans;
FJ705205
k__Plantae;p__Chlorophyta;c__Trebouxiophyceae;o__Trebouxiales;f__Trebouxiaceae;g__Trebouxia;s__Trebouxia_decolorans;
FJ705184
k__Plantae;p__Chlorophyta;c__Trebouxiophyceae;o__Trebouxiales;f__Trebouxiaceae;g__Trebouxia;s__Trebouxia_decolorans;
JX036160
k__Plantae;p__Chlorophyta;c__Trebouxiophyceae;o__Trebouxiales;f__Trebouxiaceae;g__Trebouxia;s__Uncultured_Trebouxia;
KF907553
k__Plantae;p__Chlorophyta;c__Trebouxiophyceae;o__Trebouxiales;f__Trebouxiaceae;g__Trebouxia;s__Trebouxia_jamesii;
KT827678 k__Plantae;p__Chlorophyta;c__Trebouxiophyceae;o__Trebouxiales;f__Trebouxiaceae;g__Trebouxia;s__Trebouxia_jamesii;
KT827707
k__Plantae;p__Chlorophyta;c__Trebouxiophyceae;o__Trebouxiales;f__Trebouxiaceae;g__Trebouxia;s__Trebouxia_jamesii;
JX675737
k__Plantae;p__Chlorophyta;c__Ulvophyceae;o__Trentepohliales;f__Trentepohliaceae;g__Trentepohlia;s__Trentepohlia_iolithus;
JX675738
k__Plantae;p__Chlorophyta;c__Ulvophyceae;o__Trentepohliales;f__Trentepohliaceae;g__Trentepohlia;s__Trentepohlia_arborum;
JX675736
k__Plantae;p__Chlorophyta;c__Ulvophyceae;o__Trentepohliales;f__Trentepohliaceae;g__Trentepohlia;s__Trentepohlia_umbrina;
JX675731
k__Plantae;p__Chlorophyta;c__Ulvophyceae;o__Trentepohliales;f__Trentepohliaceae;g__Trentepohlia;s__Trentepohlia_sp;
JQ617945
k__Plantae;p__Chlorophyta;c__Ulvophyceae;o__Trentepohliales;f__Trentepohliaceae;g__Trentepohlia;s__Trentepohlia_sp;

KC489149
k__Plantae;p__Chlorophyta;c__Ulvophyceae;o__Trentepohliales;f__Trentepohliaceae;g__Printzina;s__Printzina_cf_lagenifera;
KC489150
k__Plantae;p__Chlorophyta;c__Ulvophyceae;o__Trentepohliales;f__Trentepohliaceae;g__Printzina;s__Printzina_lagenifera;
KC489134 k__Plantae;p__Chlorophyta;c__Ulvophyceae;o__Trentepohliales;f__Trentepohl-iaceae;g__Printzina;s__Printzina_sp;
KY559106
k__Plantae;p__Chlorophyta;c__Trebouxiophyceae;o__Trebouxiales;f__Trebouxiaceae;g__Asterochloris;s__Asterochloris_irregu-laris;
KP031547
k__Plantae;p__Chlorophyta;c__Trebouxiophyceae;o__Trebouxiales;f__Trebouxiaceae;g__Asterochloris;s__Asterochlo-ris_cf_glomerata;
KP257384
k__Plantae;p__Chlorophyta;c__Trebouxiophyceae;o__Trebouxiales;f__Trebouxiaceae;g__Asterochloris;s__Asterochloris_medi-terranea;
KP318674
k__Plantae;p__Chlorophyta;c__Trebouxiophyceae;o__Trebouxiales;f__Trebouxiaceae;g__Asterochloris;s__Asterochloris_lobo-phora;
KP318671
k__Plantae;p__Chlorophyta;c__Trebouxiophyceae;o__Trebouxiales;f__Trebouxiaceae;g__Asterochloris;s__Asterochloris_echi-nata;
KP318670
k__Plantae;p__Chlorophyta;c__Trebouxiophyceae;o__Trebouxiales;f__Trebouxiaceae;g__Asterochloris;s__Asterochlo-ris_friedlii;
KP318669
k__Plantae;p__Chlorophyta;c__Trebouxiophyceae;o__Trebouxiales;f__Trebouxiaceae;g__Asterochloris;s__Asterochlo-ris_woessiae;
KP318664
k__Plantae;p__Chlorophyta;c__Trebouxiophyceae;o__Trebouxiales;f__Trebouxiaceae;g__Asterochloris;s__Asterochlo-ris_gaertneri;
KY981701
k__Plantae;p__Chlorophyta;c__Trebouxiophyceae;o__Trebouxiales;f__Trebouxiaceae;g__Myrmecia;s__Myrmecia_israelensis;
KY981700
k__Plantae;p__Chlorophyta;c__Trebouxiophyceae;o__Trebouxiales;f__Trebouxiaceae;g__Myrmecia;s__Myrmecia_israelensis;

List 2. Compounds screened using the software UNIFI scientific information system (Waters):

1,3-Dihydro-4,6-dihydroxy-3-oxo-5-isobenzofurancarboxaldehyde
1,7-Dibenzofurandicarboxylic acid, 3,9-dimethoxy-, dimethyl ester
11H-Dibenzo[b,e][1,4]dioxepin-7-carboxylic acid, 3,8-dimethoxy-11-oxo-6-pentyl-1-valeryl-, methyl ester
1-O-Methylfragilin
2,4,5-Trichloro-1,3-dihydroxy-6-methoxy-8-methyl-9H-xanthen-9-one
2,4,5-Trichlorolichexanthone
2,4,7-Trichloro-3,8-dihydroxy-6-methoxy-1-methyl-9H-xanthen-9-one
2,4-Dichlorolichexanthone
2,4-Dichloronorlichexanthone
2,4'-Di-O-methylnorsekikaic acid
2,5,7-Trichlorolichexanthone
2,5-Dichloro-3,8-dihydroxy-6-methoxy-1-methyl-9H-xanthen-9-one
2,5-Dichlorolichexanthone
2,5-Dichloronorlichexanthone
2,7-Dichlorolichexanthone
2,7-Dichloronorlichexanthone
2-Chloro-1-hydroxy-3,6-dimethoxy-8-methyl-9H-xanthen-9-one
2-Chloro-8-hydroxy-3,6-dimethoxy-1-methyl-9H-xanthen-9-one
2-Chloronorlichexanthone
2-Hydroxy-3-[(1-oxohexadecyl)oxy]propyl 6-deoxy-6-sulfo-α-D-glucopyranoside
2-Hydroxychrysophanol
2'-O-Methylanziaic acid
2-O-Methylconfluentic acid
2-O-Methyldivaricatic acid
2'-O-Methylevernic acid
2-O-Methylhiascic acid
2'-O-Methylhyperphyllinic acid A
2'-O-Methylhyperphyllinic acid B
2'-O-Methylimbricaric acid
2'-O-Methylmicrophyllinic acid
2'-O-Methylperlatolic acid
2-O-Methylperlatolic acid
2'-O-Methylphysodic acid
2-O-Methylsekikaic acid
2-O-Methylstenosporic acid
2''-O-Methyltenuiorin
2'-O-Methyltenuiorin
3,5-Dichloro-2'-O-methylanziac acid
3,5-Dichloro-2'-O-methylnorstenosporic acid
3,5-Dichloro-4-O-demethylplanaic acid
3,7-Dimethoxy-1,9-dibenzofurandicarboxylic acid
3-0-Methylasemone
3-Chlorodivaricatic acid
3-O-Methylthiophanic acid
4,4’-Disolorinic acid
4,5-Dichloronorlichexanthone
4,5-Di-O-methylhiascic acid
4,7-Dichloronorlichexanthone
4-0-Demethylglomellic acid
4-0-Demethylloxodellic acid
4-Chloro-1,6-dihydroxy-3-methoxy-8-methyl-9H-xanthen-9-one
4-Chloronorlichexanthone
4-Dechlorothiomelin
4-Methoxyphthalic acid
4-O-Demethylbarbatic acid
4-O-Demethyldiffractaic acid
4-O-Demethylglomelliferic acid
4-O-Demethylimbricaric acid
4-O-Demethylmicrophyllinic acid
4-O-Demethylplanaic acid

4'-O-Demethylsekikaic acid
4-O-Demethylsphaerophorin
4-O-Demethylstenosporic acid
4-O-Methylcryptochlorophaeic acid
4-O-Methylcryptochloropheic acid
4-O-Methylgyrophoric acid
4-O-Methylisocryptochlorophaeic acid
4-O-Methylolivetoric acid
4-O-Methylphysodic acid
4-O-Methylsuperolivetoric acid
5,7-Dichloronorlichexanthone
5,7-Dideoxyquercetin
5-Chloro-2'-O-methylanziaic acid
5-Chloroemodin
5-Chlorovirensic acid
5-O-Methylhiascic acid
6,8,1'-Tri-O-methyl averantin
6-0-Methylthiophanic acid
6-Heptyl-3,8-dihydroxy-1-methyl-11-oxo-11H-dibenzo[b,e][1,4]dioxepin-7-carboxylic acid
6-O-Methylaverantin
6-O-Methylthiophanic acid
7,7'-Dichlorohypericin
7-Chloro-1,3-dihydroxy-6-methoxy-8-methylxanthone
7-Chloro-1,6-di-O-methylemodin
7-Chloro-1-O-methylemodin
7-Chloroemodin
7-Chlorolichexanthone
7-Chloronorlichexanthone
7H-Isobenzofuro[4,5-b][1,4]benzodioxepin-3,7(1H)-dione, 4,10-dimethoxy-5,8,11-trimethyl-
7-O-Methylemodic acid
8-O-Methylaverythin
8-O-Methylthiomelin
Alatinone
Alectorialic acid
Alectorialin
Alectoronic acid
Alectosarmentin
alpha Acetylhypoconstictic acid
Amidepsine D
Anthraquinone, 2-chloro-1,6,8-trihydroxy-3-methyl-
Anziaic acid
Aphthosin
Apigenin
Argopsin
Arthoniaic acid
Arthonin
Arthothelin
Asemone
Aspicilin
Atranorin
Averantin
Averythrin
Azaleatin
Baeomycesic acid
Baicalein
Barbatic acid
Barbatolic acid
Barbatolin
Benzoic acid, 2-
Benzoic acid, 2'-heptyl-4',6'-dihydroxy-4-methoxy-6-methyl-2,3'-oxydi-, δ-lactone
Benzoic acid, 2-heptyl-6-hydroxy-4-methoxy-, 4-carboxy-3-heptyl-5-methoxyphenyl ester
Benzoic acid, 3-

Benzoic acid, 3-chloro-2-hydroxy-4-methoxy-6-pentyl-, 4-carboxy-3-hydroxy-5-pentylphenyl ester
Boninic acid
Buellolide
Caloploicin
Calycin
Canarione
Capillarisin
Chloroatranorin
Colensoic acid
Coneuplectin
Confluentic acid
Congrayanic acid
Conloxodin
Connorstictic acid
Conorlobaridone
Consalazinic acid
Constictic acid
Constipatic acid
Contortin
Crustinic acid
Cryptochlorophaeic acid
Cryptostictic acid
Deacetylroccellin
Decarboxythamnolic acid
Dechlorodiploicin
Demethylchodatin
Demethylpsoromic acid
Deoxyerythrolaccin
Diacetylgraciliformin
Didymic acid
Diffractaic acid
Diploicin
Diploschistesic acid
Divaricatic acid
Echinocarpic acid
Emodic acid
Emodin
Endocrocin
Epanorin
Epiphorellic acid 1
Epiphorellic acid 2
Eriodermin
Erythrin
Erythroglaucin
Ethyl everninate
Ethyl haematommate
Eugenitin
Eugenitol
Eumitrin A1
Eupafolin
Euplectin
Evernic acid
Evernin
Everninic acid
Fallacinal
Ferulic acid
Flavipin
Fragilin
Fumarprotocetraric acid
Funiculosin
Fusarubin
Galangin

Galbinic acid
Gangaleoidin
Genistein
Glaucophaeic acid
Glomellic acid
Glomelliferic acid
Glomelliferonic acid
Glomellonic acid
Grayanic acid
Gyrophoric acid
Haematommone
Hematommic acid
Hiascic acid
Homosekikaic acid
Hydroxyemodin
Hyperconfluentic acid
Hyperhomosekikaic acid
Hyperlatolic acid
Hypoconstictic acid
Hypoprotocetraric acid
Hyposalazinic acid
Hypostictic acid
Hypothallin
Hypothamnolic acid
Imbricaric acid
Insignin
Isoarthothelin
Isohematommic acid
Isohyperlatolic acid
Isonotatic acid
Isoobtusatic acid
Isophysodic acid
Isorhamnetin
Isosubpicrolichenic acid
Isousnic acid
Isovicanicin
Lanosterol
Lasallic acid
Lecanoric acid
Lecideoidin
Leoidin
Leprapinic acid
Lepraric acid
Leprolomin
Lichexanthone
Lividic acid
Lobaric acid
Loxodellic acid
Loxodin
Lucidin
Lusitanic acid
Malonprotocetraric acid
Melacarpic acid
Menegazziaic acid
Merochlorophaeic acid
Methyl 2-O-methyllecanorate
Methyl 3,5-dichlorolecanorate
Methyl 4-O-demethylbarbatate
Methyl barbatate
Methyl evernate
Methyl gyrophorate
Methyl haematommate

Methyl lecanorate
Methyl olivetolate
Methyl orsellinate
Methyl β-orsellinate
Miriquidic acid
Montagnetol
Moretenone
Morindone
m-Scrobiculin
m-Scrobiculin
Neosartorin
Nephroarctin
Neuropogonin A
Norcolensoic acid
Nordivaricatic acid
Norgangaleoidin
Norlichexanthone
Norlobaric acid
Norlobaridone
Norlobariol
Normiriquidic acid
Nornotatic acid
Norperistictic acid
Norsolorinic acid
Norstictic acid
Norwogonin
Notatic acid
Obtusatic acid
Olivetolic acid
Olivetoric acid
O-Methylhypoprotocetraric acid
Orsellinic acid
Ovoic acid
Oxyphysodic acid
Paludosic acid
Pannaric acid
Pannaric acid 2-methyl ester
Pannaric acid 6-methyl ester
Pannarin
Parietin
Parietinic acid
Pedalitin
Perlatolic acid
Phenarctin
Physodalic acid
Physodic acid
Picrolichenic acid
Pinastric acid
Placodiolic acid
Planaic acid
Porphyrilin
Porphyrillic acid
Protocetraric acid
Protosiphulin
Pseudocyphellarin A
Pseudocyphellarin B
Pseudoplacodiolic acid
Psoromic acid
Pulvinic acid
Pulvinic dilactone
Pulvinone
Ramalic acid

Rangiformic acid
Resokaempferol
Rhabarberone
Rhamnetin
Rhizinonic acid
Rhizocarpic acid
Rhodocladonic acid
Salizinic acid
Scabrosin
Schizopeltic acid
Secalonic acid A
Secalonic acid B
Secalonic acid C
Sekikaic acid
Sexangularetin
Siphulellic acid
Siphulin
Skyrin
Solorinic acid
Sordidone
Sphaerophorin
Squamatic acid
Stenosporic acid
Stenosporonic acid
Stictic acid
Stictinic acid
Strepsilin
Subdidymic acid
Submerochlorophaeic acid
Subpaludosic acid
Subpicrolichenic acid
Substictic acid
Succinprotocetraric acid
Sulphuretin
Superconfluentic acid
Superlatolic acid
Superpicrolichenic acid
Sydowinin B
Tamaraxetin
Teloschistin
Tenuiorin
Tetra-O-methylaverythrin
Thamnolic acid
Thiomelin
Thiophanic acid
Thiophaninic acid
threo-Hex-2-enaric acid, 2-deoxy-2-(4-methoxyphenyl)-5-C-phenyl-, 1,4-lactone, 6-methyl ester
Thunberginol A
Thuringione
Umbilicaric acid
Usnic acid
Valsarin
Variolaric acid
Wedelolactone
Verrucigeric acid
Vicanicin
Vinetorin
Virensic acid
Vittatolic acid
Wrightiin
Vulpinic acid
Xanthorin

Xerocomic acid
α-Collatolic acid
